# Author Correction: Design-rules for stapled peptides with in vivo activity and their application to Mdm2/X antagonists

**DOI:** 10.1038/s41467-025-56965-w

**Published:** 2025-02-13

**Authors:** Arun Chandramohan, Hubert Josien, Tsz Ying Yuen, Ruchia Duggal, Diana Spiegelberg, Lin Yan, Yu-Chi Angela Juang, Lan Ge, Pietro G. Aronica, Hung Yi Kristal Kaan, Yee Hwee Lim, Andrea Peier, Brad Sherborne, Jerome Hochman, Songnian Lin, Kaustav Biswas, Marika Nestor, Chandra S. Verma, David P. Lane, Tomi K. Sawyer, Robert Garbaccio, Brian Henry, Srinivasaraghavan Kannan, Christopher J. Brown, Charles W. Johannes, Anthony W. Partridge

**Affiliations:** 1 MSD International, Singapore, 138665 Singapore; 2 Merck & Co., Inc., Kenilworth, NJ 07033 USA; 3 Institute of Sustainability for Chemicals, Energy and Environment, Agency for Science, Technology and Research (ASTAR), Singapore, 138665 Singapore; 4 Merck & Co., Inc., Boston, MA 02115 USA; 5 Department of Surgical Sciences, Uppsala University, Uppsala, Sweden; 6 Bioinformatics Institute, Agency for Science, Technology and Research (ASTAR), Singapore, 138671 Singapore; 7 Merck & Co., Inc., West Point, PA 19486 USA; 8 Department of Immunology, Genetics and Pathology, Uppsala University, Uppsala, Sweden; 9 Institute of Molecular and Cell Biology, Singapore, 138673 Singapore; 10 EPOC Scientific LLC, Stoneham, MA 02180 USA; 11 Present Address: Genentech, South San Francisco, CA 94080 USA

Correction to: *Nature Communications* 10.1038/s41467-023-43346-4, published online 12 January 2024

In the version of the article initially published, in the “Selection of staple position” section of the Discussion, the sentence “Although many staple types exist, our suggestion is to limit the initial analogs to a single chemistry: olefin staples resulting from ring closing metathesis of (S)-2-(4’-pentenyl)alanine and (R)-2-(7’-octenyl)alanine at the i and i + 7 positions, respectively” should have read “Although many staple types exist, our suggestion is to limit the initial analogs to a single chemistry: olefin staples resulting from ring closing metathesis of (R)-2-(7’-octenyl)alanine and (S)-2-(4’-pentenyl)alanine at the i and i + 7 positions, respectively” and has now been corrected in the HTML and PDF versions of the article.

