# Supplementary material for: Design-rules for stapled peptides with in vivo activity and their application to Mdm2/X antagonists
Source: Nat Commun. 2024 Jan 12;15:489. doi: 10.1038/s41467-023-43346-4 (PMC10786919; doi:10.1038/s41467-023-43346-4)
Supplement: Supplementary file 1 — Supplementary Information [file 41467_2023_43346_MOESM1_ESM.pdf]

Supplementary Information for:

Design-Rules for Stapled Peptides with in vivo Activity and their Application to Mdm2/X antagonists

Arun Chandramohan[1], Hubert Josien[2], Tsz Ying Yuen[3], Ruchia Duggal[4], Diana Spiegelberg[5], Lin Yan[2], Yu-Chi Angela Juang[1], Lan Ge[2], Pietro G. Aronica[6], Hung Yi Kristal Kaan[1], Yee Hwee Lim[3], Andrea Peier[2], Brad Sherborne[2], Jerome Hochman[7], Songnian Lin[2], Kaustav Biswas[4], Marika Nestor[8], Chandra S Verma[6], David P. Lane[9], Tomi K. Sawyer[4], Robert Garbaccio[2], Brian Henry[1,*], Srinivasaraghavan Kannan[6,*], Christopher J. Brown[9,*], Charles W. Johannes[3,*], Anthony William Partridge[1,ψ,*]

[1] MSD International, Singapore 138665, Singapore.

[2] Merck & Co., Inc., Kenilworth, New Jersey 07033, United States.

[3] Institute of Sustainability for Chemicals, Energy and Environment, Agency for Science, Technology and Research (ASTAR), 8 Biomedical Grove, #07, Neuros Building, Singapore 138665

[4] Merck & Co., Inc., Boston, Massachusetts 02115, United States.

[5] Department of Surgical Sciences, Uppsala University, Sweden

[6] Bioinformatics Institute, Agency for Science, Technology and Research (ASTAR), 30 Biopolis Street, #07-01 Matrix, Singapore 138671, Singapore

[7] Merck & Co., Inc., West Point, Pennsylvania, 19486, United States

[8] Department of Immunology, Genetics and Pathology, Uppsala University, Uppsala, Sweden.

[9] Institute of Molecular and Cell Biology, Singapore, 138673, Singapore.

[ψ] Current Address: Genentech, South San Francisco, CA

[*] To whom correspondence should be addressed: brian.henry3@msd.com, raghavk@bii.a-star.edu.sg, CJBrown@imcb.a-star.edu.sg, cwjohannes@gmail.com, partridge.anthony@gene.com

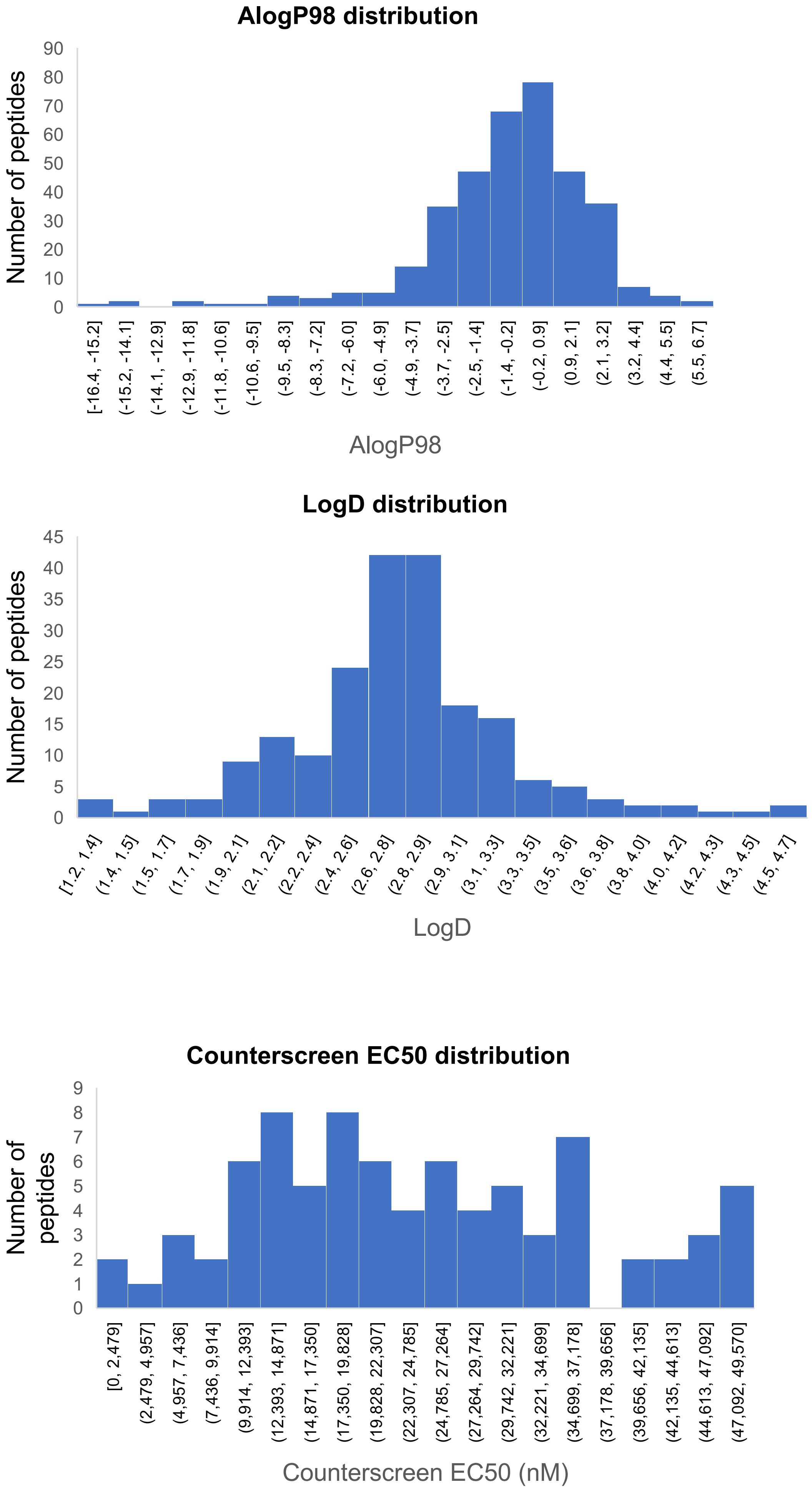

Supplementary Figure 1: Library of ATSP-7041 analogs have diverse values for polarity(AlogP98 and LogD), Counterscreen potency, LDH release activity, p53 activation, and biochemical binding

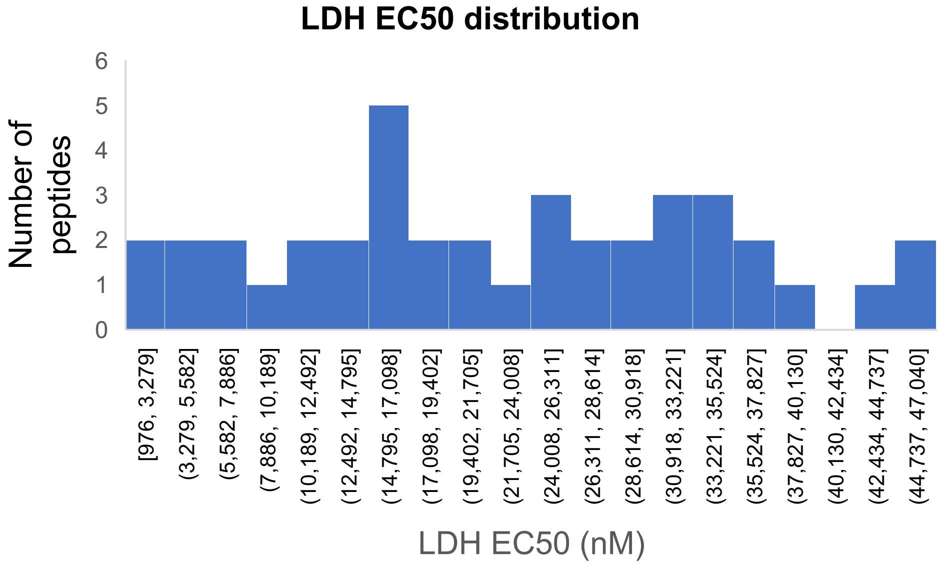

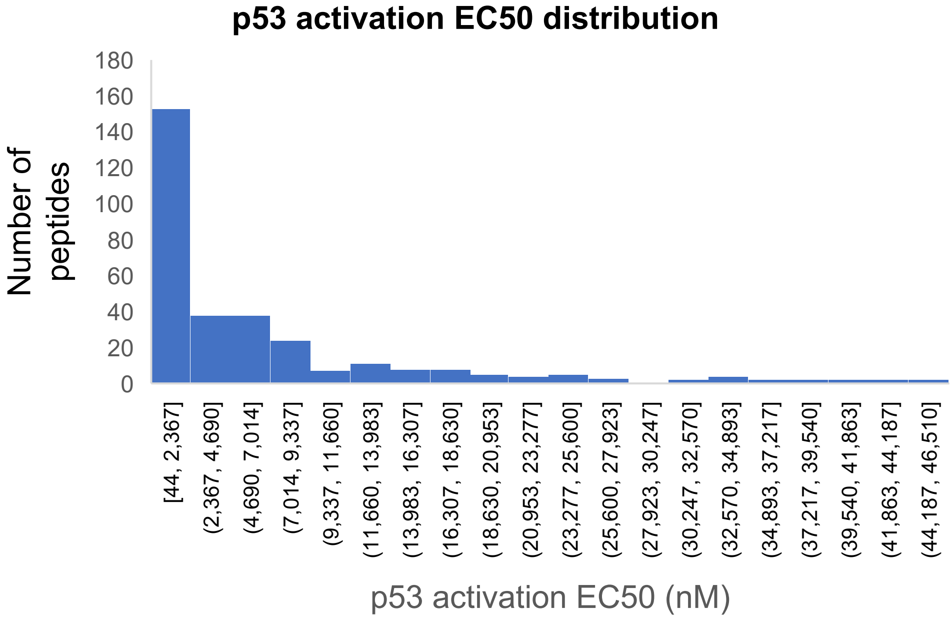

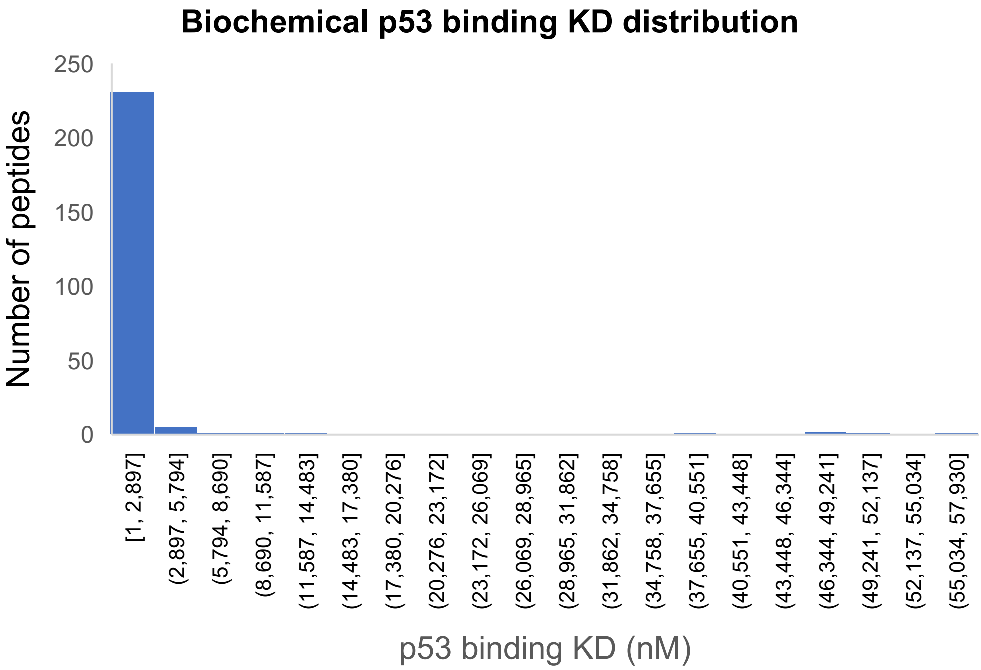

Supplementary Figure 1 continued: Library of ATSP-7041 analogs have diverse values for polarity(AlogP98 and LogD), Counterscreen potency, LDH release activity, p53 activation, and biochemical binding

A)

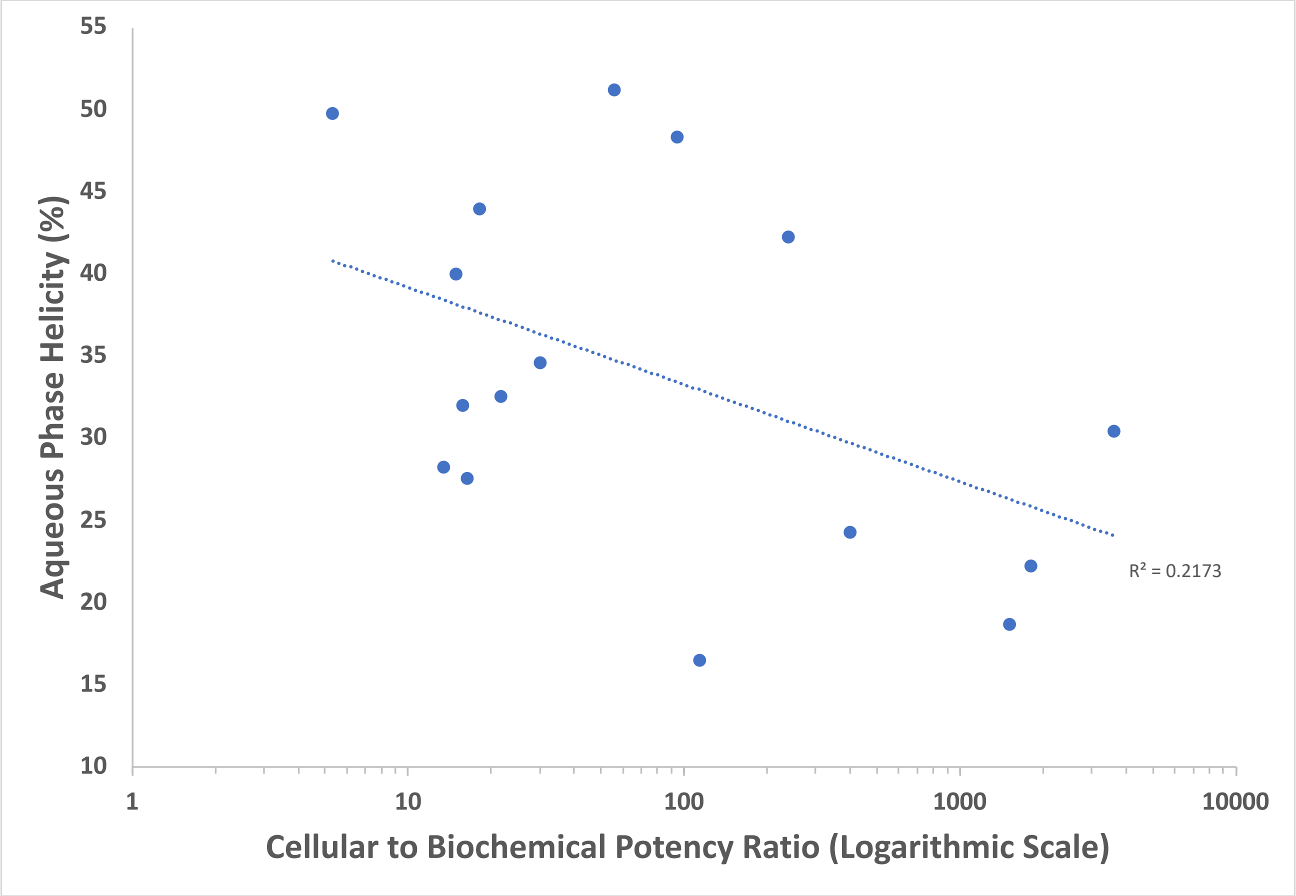

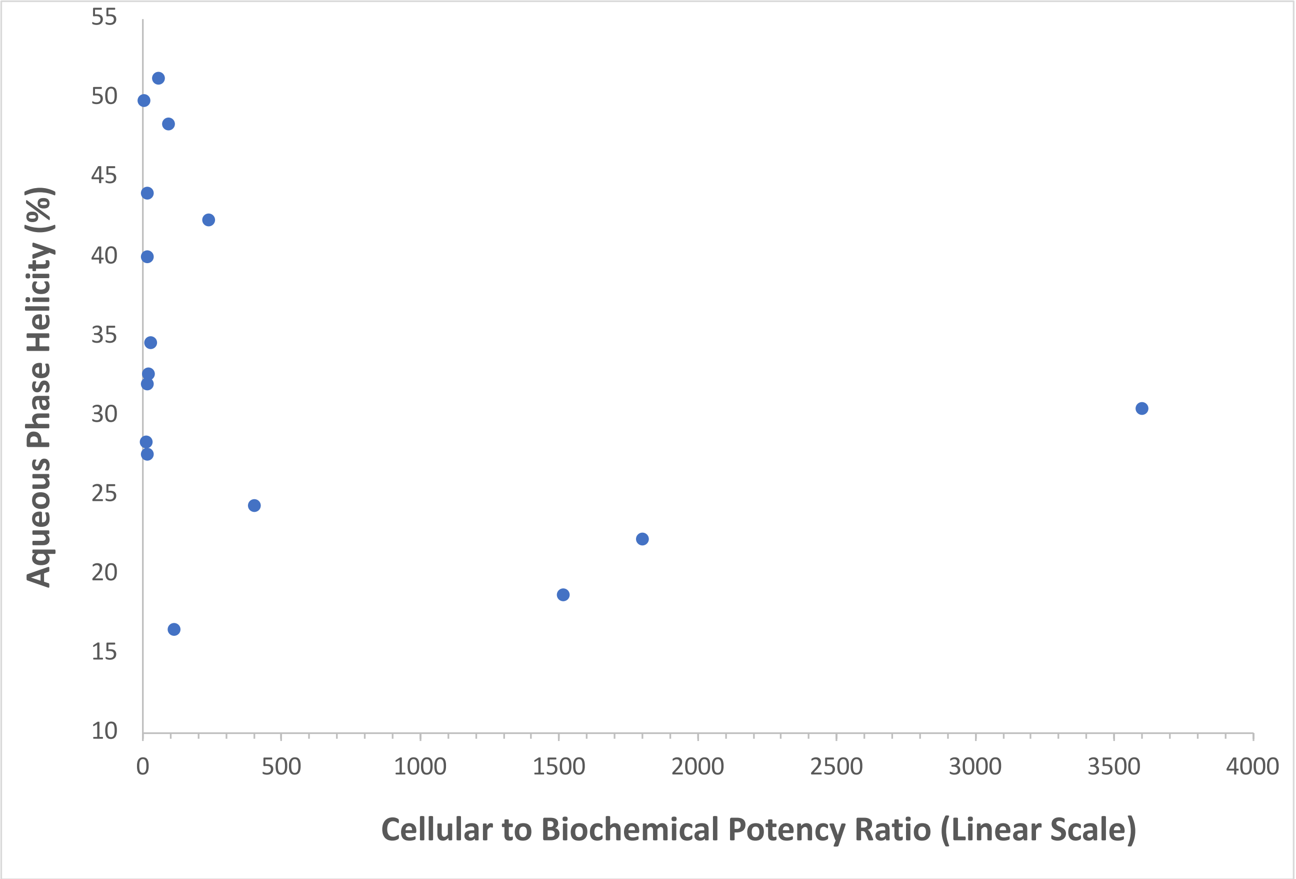

Supplementary Figure 2: Azide-ATSP-7041 analogs demonstrated weak correlations for between their aqueous phase helicity and cellular to biochemical ratios (A) Nanoclick activities and logD values (B) and a moderate correlation between logD values and cellular activities (C)

B

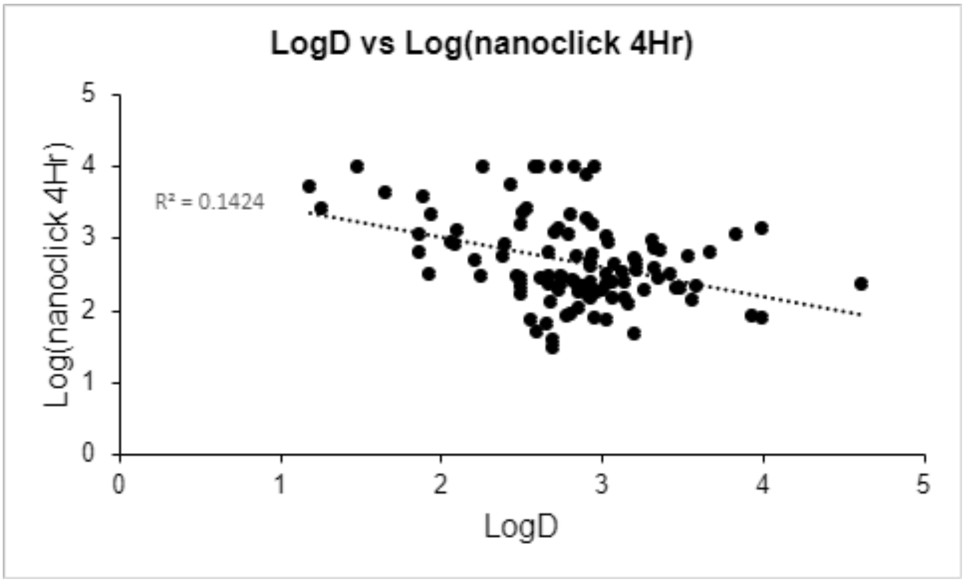

C

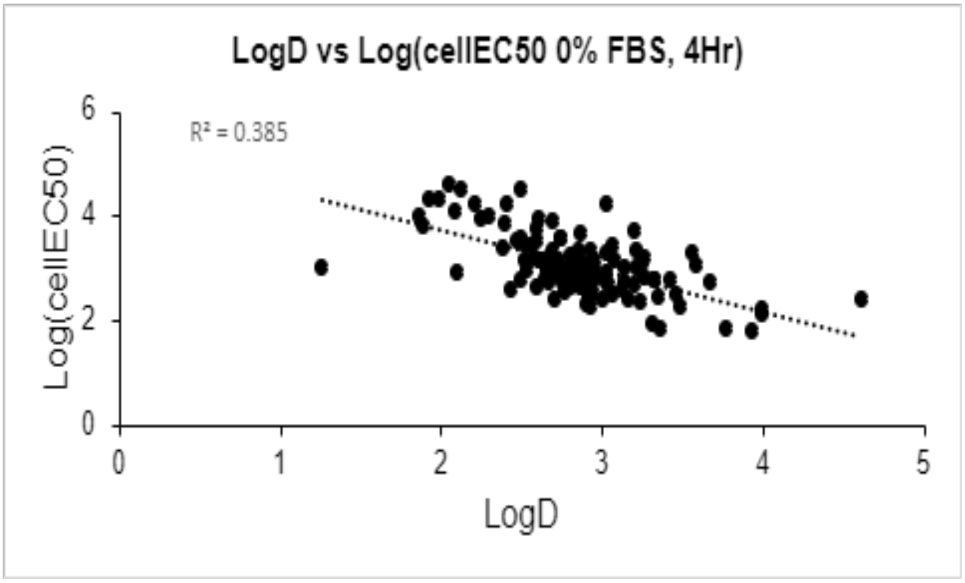

Supplementary Figure 2 cont: Azide-ATSP-7041 analogs demonstrated weak correlations for between their aqueous phase helicity and cellular to biochemical ratios (A) Nanoclick activities and logD values (B) and a moderate correlation between logD values and cellular activities (C)

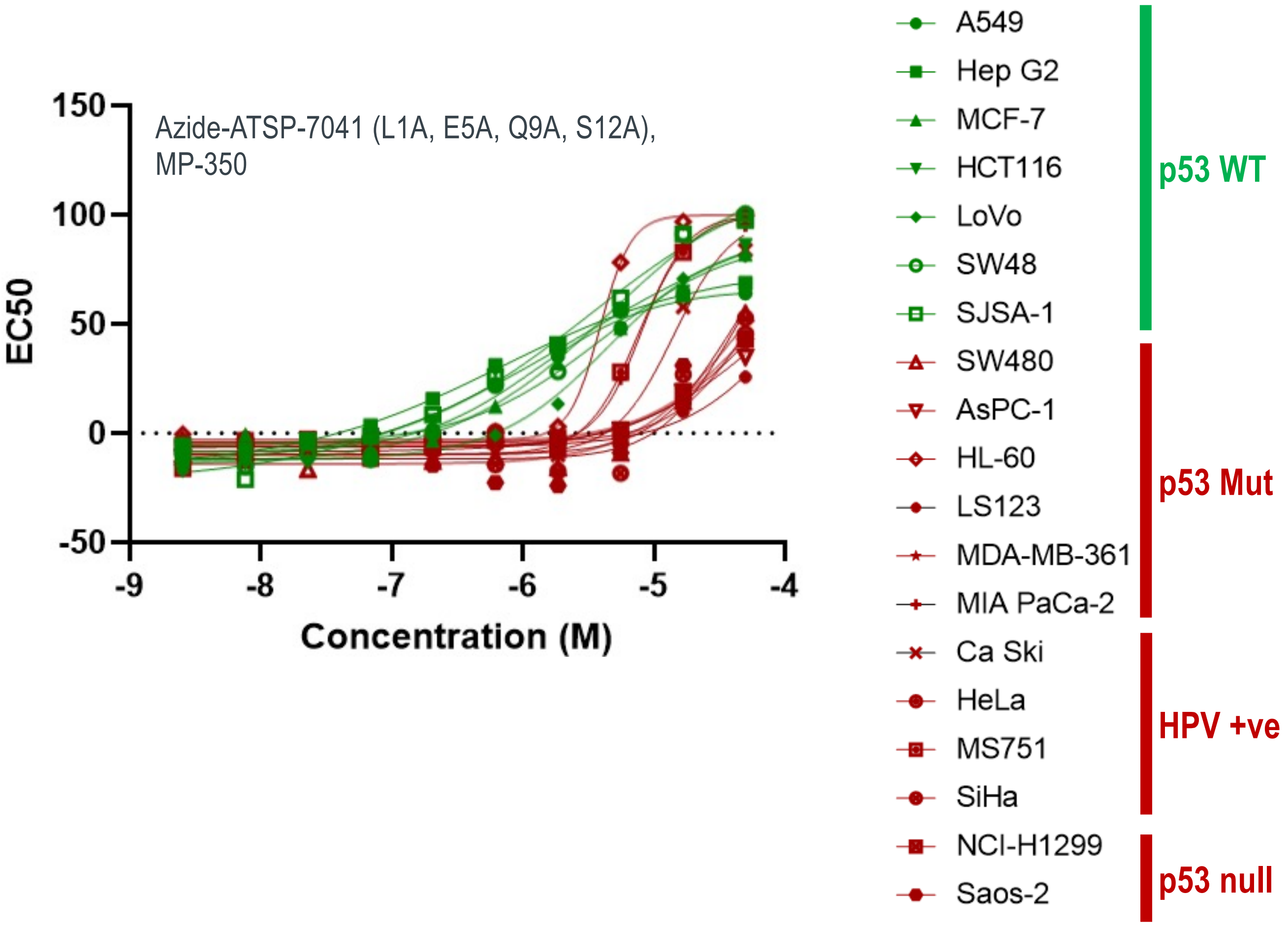

Supplementary Figure 3: A highly apolar azide-ATSP-7041 analog with alanine substitutions at positions L1, E5, Q9, and S12 (MP-350) shows cellular proliferation activities in p53 deficient cell lines (i.e., p53 null, mutant, or p53-depleted through HPV infection, red) that overlap with those in cell lines that are p53 wild-type (green), indicating significant off-target toxicity

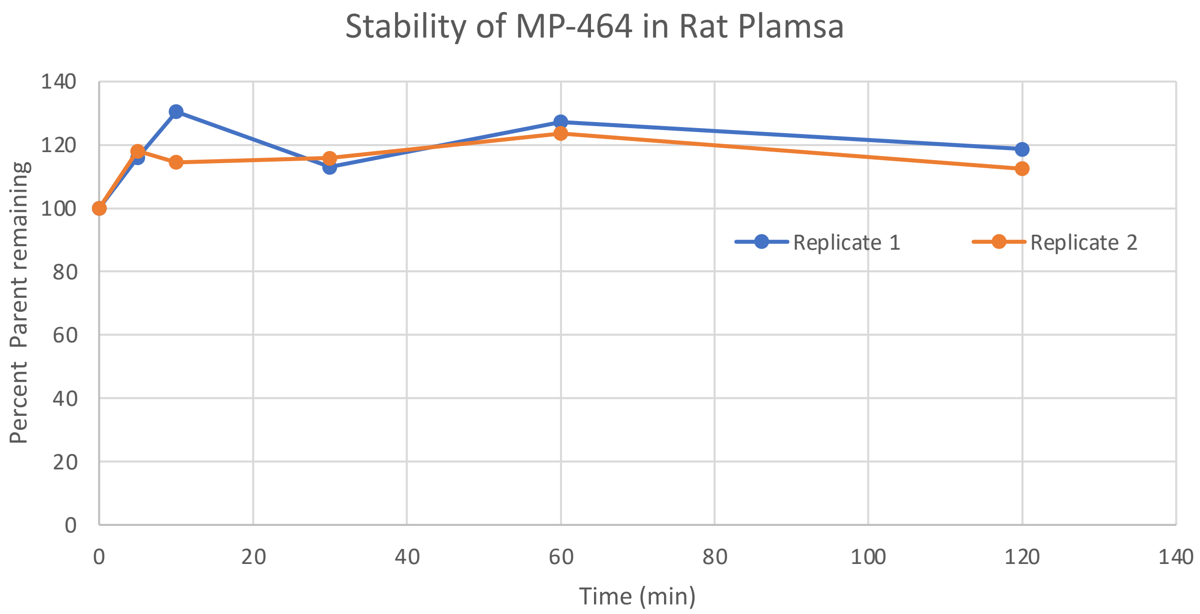

Supplementary Figure 4: Stability of MP-464 in Rat Plasma

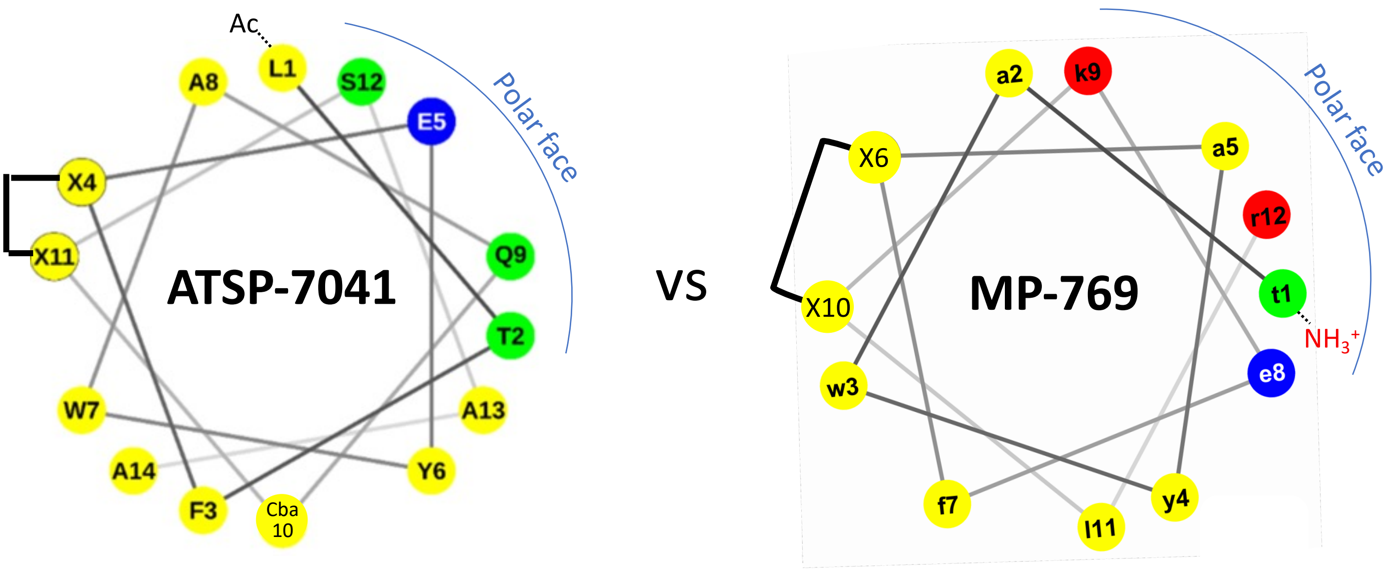

Supplementary Figure 5: Comparison of amphipathicity of all-L (ATSP-7041) and all-D (MP-769) stapled peptides. Residue in yellow are apolar, residues in green are polar uncharged, residues in blue are anionic, and residues in red are cationic

Supplementary Table 1: Summary of activities for azide-ATSP-7041 variant peptides (see Supplementary Data 1 for peptide sequences). Cellular assays (TETCHO, LDH, p53 reporter) were done with 0% serum and measured at the 4-hour time point. P.B. = poorly behaved in solubility assay conditions, result inconclusive. N.D. = No data available.

**Peptide ID**	**Trivial Name**	**Net Charge**	**Solubility (μM)**	**TETCHO: Counter-screen EC50 (μM)**	**LDH: Cytotoxicity assay EC50 (μM)**	**p53 reporter assay: EC50 (μM)**	**FP binding K_D (nM)**
MP-081	AZIDE-ATSP-7041	-1	105	>50	>50	0.49	5.0
MP-952	AZIDE-ATSP-7041 (L1A)	-1	109	>50	>50	0.78	10.7
MP-953	AZIDE-ATSP-7041 (T2A)	-1	186	>50	>50	1.0	80.5
MP-892	AZIDE-ATSP-7041 (F3A)	-1	101	>50	>50	>50	13240
MP-954	AZIDE-ATSP-7041 (E5A)	0	P.B.	>50	>50	0.28	47.7
MP-893	AZIDE-ATSP-7041(W7A)	-1	51	18.4	15.0	20.7	4595
MP-955	AZIDE-ATSP-7041 (Q9A)	-1	86.5	>50	>50	0.36	54.3
MP-894	AZIDE-ATSP-7041 (CBA10 TO A)	-1	93	7.7	>50	14.4	114.7
MP-8956	AZIDE-ATSP-7041 (S12A)	-1	108	>50	>50	0.34	36.2
MP-546	AZIDE-ATSP-7041 (L1K)	0	2	>50	>50	1.6	3.8
MP-547	AZIDE-ATSP-7041 (T2K)	0	3	>50	>50	2.1	5.4
MP-548	AZIDE-ATSP-7041 (E5K)	0	5	24.0	12.2	2.7	115.1
MP-549	AZIDE-ATSP-7041 (A8K)	0	4.5	>50	>50	3.6	11.1
MP-550	AZIDE-ATSP-7041 (Q9K)	0	4	>50	>50	1.4	8.2
MP-551	AZIDE-ATSP-7041 (S12K)	0	2	34.5	>50	1.8	39.7

MP-552	AZIDE-ATSP-7041 (L1R)	0	2	>50	>50	1.8	62.9
MP-553	AZIDE-ATSP-7041 (T2R)	0	P.B.	27.1	>50	2.1	30.6
MP-554	AZIDE-ATSP-7041 (E5R)	+1	P.B.	16.7	30.4	2.3	33.0
MP-555	AZIDE-ATSP-7041 (A8R)	0	2	>50	>50	2.3	69.4
MP-556	AZIDE-ATSP-7041 (Q9R)	0	1	14.3	>50	1.0	45.9
MP-557	AZIDE-ATSP-7041 (S12R)	0	P.B.	>50	>50	1.3	78.7
MP-896	AZIDE-ATSP-7041 (L1hR)	0	2.5	>50	>50	0.88	8.6
MP-897	AZIDE-ATSP-7041 (T2hR)	0	9	>50	>50	0.78	2.8
MP-898	AZIDE-ATSP-7041 (E5hR)	+1	2	>50	12.4	1.9	35.5
MP-899	AZIDE-ATSP-7041 (A8hR)	0	2	>50	>50	1.6	N.D.
MP-900	AZIDE-ATSP-7041 (Q9hR)	0	10	>50	>50	0.81	28.5
MP-901	AZIDE-ATSP-7041 (S12hR)	0	P.B.	>50	>50	1.1	5.0
MP-881	AZIDE-ATSP-7041 (A13hR)	0	2	>50	>50	0.89	42.5
MP-902	AZIDE-ATSP-7041 (A14hR)	0	6	>50	>50	0.90	8.6
MP-168	AZIDE-ATSP-7041 (L1 TO AIB)	-1	79.5	>50	>50	0.49	16.1
MP-169	AZIDE-ATSP-7041 (T2 TO AIB)	-1	61.5	>50	>50	2.9	231.4
MP-170	AZIDE-ATSP-7041 (A8 TO AIB)	-1	92.5	>50	>50	1.5	47.6
MP-171	AZIDE-ATSP-7041 (Q9 TO AIB)	-1	34	>50	>50	0.25	36.6
MP-172	AZIDE-ATSP-7041 (S12 TO AIB)	-1	140	>50	13.4	0.25	22.7

MP-257	AZIDE-ATSP-7041 (A13 TO AIB)	-1	25	>50	4.5	0.49	15.2
MP-173	AZIDE-ATSP-7041 (L1H)	-0.5	5	21.7	>50	4.2	0.7
MP-174	AZIDE-ATSP-7041 (T2H)	-0.5	1	5.9	>50	8.1	64.2
MP-175	AZIDE-ATSP-7041 (E5H)	+0.5	2	10.6	>50	0.62	21.1
MP-496	AZIDE-ATSP-7041 (A8H)	-0.5	20	2.8	>50	1.6	22.7
MP-176	AZIDE-ATSP-7041 (Q9H)	-0.5	2	24.4	>50	9.3	9.6
MP-177	AZIDE-ATSP-7041 (S12H)	-0.5	6.5	12.0	>50	4.3	4.5
MP-178	AZIDE-ATSP-7041 (L1E)	-2	169	>50	>50	3.3	1.9
MP-179	AZIDE-ATSP-7041 (T2E)	-2	102.5	>50	>50	33.2	42.4
MP-843	AZIDE-ATSP-7041 (F3E)	-2	151	>50	>50	>50	11220
MP-842	AZIDE-ATSP-7041 (Y6E)	-2	157	>50	>50	>50	5.1
MP-956	AZIDE-ATSP-7041 (W7E)	-2	175	>50	>50	>50	>101000
MP-180	AZIDE-ATSP-7041 (A8E)	-2	93	>50	>50	0.37	3.2
MP-181	AZIDE-ATSP-7041 (Q9E)	-2	179	>50	>50	3.7	34.6
MP-841	AZIDE-ATSP-7041 (CBA10E)	-2	162	>50	>50	>50	311.6
MP-182	AZIDE-ATSP-7041 (S12E)	-2	163	>50	>50	0.58	0.73
MP-898	AZIDE-ATSP-7041(A13E)	-2	145	>50	>50	7.0	14.2
MP-524	AZIDE-ATSP-7041 (A14E)	-2	148	>50	>50	0.86	0.7
MP-183	AZIDE-ATSP-7041 (L1W)	-1	19	>50	>50	0.54	16.5

MP-1184	AZIDE-ATSP-7041 (T2W)	-1	28.5	>50	>50	2.13	204
MP-185	AZIDE-ATSP-7041 (E5W)	-1	2	>50	>50	0.14	98.5
MP-186	AZIDE-ATSP-7041 (A8W)	-1	1	>50	>50	0.69	8.5
MP-187	AZIDE-ATSP-7041 (Q9W)	-1	3.5	>50	>50	0.52	86.0
MP-188	AZIDE-ATSP-7041 (S12W)	-1	50	>50	>50	0.62	48.0
MP-258	AZIDE-ATSP-7041 (T2V)	-1	79	30.0	>50	0.65	71.5
MP-259	AZIDE-ATSP-7041 (L1P)	-1	90.5	>50	>50	0.71	22.6
MP-8834	AZIDE-ATSP-7041 (E5P)	0	1	>50	>50	16.4	7837
MP-260	AZIDE-ATSP-7041 (Q9P)	-1	P.B.	>50	>50	3.2	45.8
MP-184	AZIDE-ATSP-7041 (S12P)	-1	P.B.	>50	>50	3.2	50.1
MP-261	AZIDE-ATSP-7041 (L1G)	-1	89	>50	>50	1.36	9.5
MP-210	AZIDE-ATSP-7041 (E5G)	0	2	>50	>50	0.28	68.2
MP-262	AZIDE-ATSP-7041 (Q9G)	-1	87	30.5	>50	0.78	19.1
MP-798	AZIDE-ATSP-7041 (S12G)	-1	80	>50	>50	0.63	12.4
MP-028	AZIDE-ATSP-7041 (Y6F)	-1	6	13.0	8.4	0.50	3.3
MP-202	AZIDE-ATSP7041 (F3 to D-PHE)	-1	143	40.8	>50	33.4	>101000
MP-7470	AZIDE-ATSP-7041 (L1 TO D-ALA)	-1	90	>50	>50	0.73	8.0
MP-7471	AZIDE-ATSP-7041 (T2 TO D-ALA)	-1	93.5	>50	>50	1.44	70.5
MP-473	AZIDE-ATSP-7041 (E5 TO D-ALA)	0	P.B.	>50	>50	0.18	11.5

MP-511	AZIDE-ATSP-7041 (A8 TO D-ALA)	-1	N.D.	>50	>50	0.92	65.4
MP-474	AZIDE-ATSP-7041 (Q9 TO D-ALA)	-1	156	>50	>50	0.32	18.5
MP-475	AZIDE-ATSP-7041 (S12 TO D-ALA)	-1	17	>50	>50	0.46	4.8
MP-030	AZIDE-ATSP-7041 (W7 TO TRIFLUOROMETHYL-PHE)	-1	P.B.	>50	>50	0.26	1.0
MP-765	AZIDE-ATSP-7041 (W7 TO 3,4-DICHLORO-PHE)	-1	P.B.	11.8	>50	0.90	5.3
MP-004	AZIDE-ATSP-7041 (W7 TO 6-FLURO-1-TRP)	-1	110	>50	23.9	0.20	1.0
MP-495	AZIDE-ATSP-7041 (L1D, E5D, A8D)	-3	78.67	>50	>50	21.3	5.2
MP-359	AZIDE-ATSP-7041 (L1N, E5N, A8N, S12N, A13N, A14N)	-1	53.67	>50	>50	32.3	15.0
MP-537	AZIDE-ATSP-7041 (L1R, Q9R, S12K)	+2	140	18.7	7.3	3.9	64.6
MP-538	AZIDE-ATSP-7041 (L1R, Y6F, L9R)	+1	5	>50	6.8	4.92	116.8
MP-539	AZIDE-ATSP-7041 (L1R, A8R, A13R)	+2	97.5	50.0	13.4	7.12	9.1
MP-878	NH2-GRSRSRRAA-AZIDE-ATSP-7041(L1A, E5A, Q9A, S12A)	+5	P.B.	6.7	32.9	0.65	49.1
MP-879	NH2-PAAKRVKLDG-AZIDE-ATSP-7041	+3	162	35.8	>50	2.4	38.7
MP-950	NH2-EEE-AZIDE-SGS-PM2-EEE	-6	118	>50	>50	>50	2.4

Supplementary Table 2 (see Supplementary Data 1 for peptide sequences): Histidine substitutions generally result in more potent and higher efficacious counterscreen activity.

**Peptide ID**	**Trivial Name**	**TETCHO Counterscreen (4 hours, 0% serum)**	
		**EC50 (μM)**	**% Effect**
MP-081	AZIDE-ATSP-7041	>50	15.0
MP-173	AZIDE-ATSP-7041 (L1H)	21.7	172.1
MP-174	AZIDE-ATSP-7041 (T2H)	5.9	277.4
MP-175	AZIDE-ATSP-7041 (E5H)	10.6	78.52
MP-176	AZIDE-ATSP-7041 (Q9H)	24.4	222.1
MP-177	AZIDE-ATSP-7041 (S12H)	12.0	234.8
MP-197	ATSP-7041 (L1H)	21.5	154.7
MP-198	ATSP-7041 (T2H)	13.1	258.7
MP-199	ATSP-7041 (E5H)	22.7	110.5
MP-200	ATSP-7041 (L9H)	9.9	48.83
MP-201	ATSP-7041 (S12H)	16.5	217.7
MP-496	AZIDE-ATSP-7041 (A8H)	2.8	34.53
MP-501	ATSP-7041 (Q8H)	18.9	216

Supplementary Table 3 (see Supplementary Data 1 for peptide sequences). P.B. = poor behaved (no value could be discerned). N.D. = No data available

**Peptide ID**	**Trivial name**	**Partition Coeff**	**AlogP98**	**TETCHO: Counterscreen**	**LDH: Cytotoxicity assay**	**p53 reporter assay: EC50 (nM)**		**FP Binding (nM)**	**Solubility**
				**0% FBS 4Hr**	**0% FBS 4Hr**	**0% FBS 4Hr**	**10% FBS 4 Hr**	**Tween-20**	
		**LogD**	**AlogP98**	**EC50 (μM)**	**EC50 (μM)**	**EC50 (μM)**		**K_D**	**(μM)**
MP-081	AZIDE-ATSP-7041	2.82	-0.054	>50	>50	0.49	2.5	5	105
MP-953	AZIDE-ATSP-7041 (T2A)	2.93	0.456	>50	>50	1.0	N.D.	80.5	186
MP-954	AZIDE-ATSP-7041 (E5A)	P.B.	1.718	>50	>50	0.28	1.4	48.7	P.B.
MP-955	AZIDE-ATSP-7041 (Q9A)	2.94	0.871	>50	>50	0.36	2.9	54.3	86.5
MP-8956	AZIDE-ATSP-7041 (S12A)	2.925	0.834	>50	>50	0.34	5.1	36.2	108
MP-957	AZIDE-ATSP-7041 (L1A,T2A)	2.695	-0.775	>50	17.1	2.2	N.D.	26.47	101
MP-958	AZIDE-ATSP-7041 (L1A,E5A)	3.13	0.486	>50	>50	0.40	N.D.	41.74	5
MP-959	AZIDE-ATSP-7041 (L1A,Q9A)	2.785	-0.36	>50	0.98	0.44	N.D.	21.14	105.5
MP-960	AZIDE-ATSP-7041 (L1A,S12A)	2.76	-0.398	>50	>50	0.52	N.D.	16.06	101.5
MP-961	AZIDE-ATSP-7041 (T2A,E5A)	P.B.	2.228	>50	>50	0.46	N.D.	272.3	P.B.
MP-962	AZIDE-ATSP-7041 (T2A,Q9A)	2.96	1.382	14.8	>50	0.90	N.D.	79.47	77.5
MP-963	AZIDE-ATSP-7041 (T2A,S12A)	2.97	1.345	35.9	>50	0.66	N.D.	37.07	100
MP-964	AZIDE-ATSP-7041 (E5A,Q9A)	3.77	2.643	>50	>50	0.072	0.84	37.99	P.B.
MP-965	AZIDE-ATSP-7041 (E5A,S12A)	P.B.	2.606	41.0	>50	0.044	0.96	30.9	P.B.
MP-693	AZIDE-ATSP-7041 (E5L, A8L)	2.925	0.834	>50	>50	0.34	5.1	36.2	<1
MP-8966	AZIDE-ATSP-7041 (Q9A,S12A)	2.92	1.76	>50	>50	0.28	4.7	45.27	111
MP-967	AZIDE-KK-ATSP-7041 (L1A,T2A,E5A,Q9A,S12A)	2.75	-0.27	18.5	16.8	14.5	N.D.	156.1	P.B.
MP-209	AZIDE-ATSP-7041 (L1A, E5A, Q9A)	3.32	1.411	>50	>50	0.089	3.0	95.99	2

MP-350	AZIDE-ATSP-7041 (L1A, E5A, Q9A, S12A)	3.367	2.3	>50	>50	0.068	0.17	53.93	2
MP-694	AZIDE-ATSP-7041 (L1Y, E5Y, A8Y)	3.22	4.428	>50	>50	1.0	3.8	371.1	9
MP-490	AZIDE-ATSP-7041 (L1V, E5V, A8V)	4	3.015	>50	>50	0.17	2.5	122.5	3
MP-492	AZIDE-ATSP-7041 (L1F, E5F, A8F)	4.21	5.154	>50	>50	0.76	27.9	N.D.	3
MP-493	AZIDE-ATSP-7041 (L1 TO D-LEU, E5A, Q9A, W7 TO 6-CLTRP, A13 TO D-ALA)	3.93	3.308	19.5	>50	0.062	0.46	16.9	<1

Supplementary Results: Peptide QC files

CHINESE PEPTIDE

Certificate of Analysis

Product Name: azide-ATSP-7041 (alkyne staple, AA(a-Me-E)AAa tail) **Barcode#:** 0955848658

Product #: 994408 **Lot #:** CU-06-00367

Sequence: Ac-Lys(N3)-BetaAla-Leu-Thr-Phe-((R)-2-amino-2-methyloct-7-ynoic acid)-Glu-Tyr-Trp-Ala-Gln-Cba-((S)-2-amino-2-methylhept-6-ynoic acid)-Ala-Ala-(alpha MethylGlu)-Ala-Ala-D_Ala-NH2 (alkyne coupling between (R)-2-amino-2-methyloct-7-ynoic acid and (S)-2-amino-2-methylhept-6-ynoic acid)

Molecular Weight: 2233.5

Mass Spectral Analysis: Electrospray “Exhibits correct MW” (see attached MS spectrogram)

HPLC Analysis: Peptide purity: 93.7% (see attached RP-HPLC chromatogram)

Solubility: 1 mg/ml in DMSO

Appearance: White lyophilized powder

Counter Ion: Ammonium salt

Cert. of Analysis Remarks:

Remarks: **Not for human use, research purpose only.**

Quality Assurance By: Hawk Zhu **Date: 06/29/21**
Quality Control Department

Chinese Peptide Company
No.69,12 Street,Hangzhou Economic & Technological Development Zone,China 310018
Tel: 86-571-86737011 86737118-8686
Fax: 86-571-86737013
Email: sales@chinesepeptide.com
Website: www.chinesepeptide.com

QC files for MP-467

Data file: D:\DATE\210629\20210629-1 2021-06-29 09-58-04\994408.D 6/29/2021 11:57:10 AM

Sample name: 994408

Description: Lot#:CU-06-00367
MW:2233.5
Sample Type:Final

Acq. method: 290-2000-Negative-
Frag 130v.M

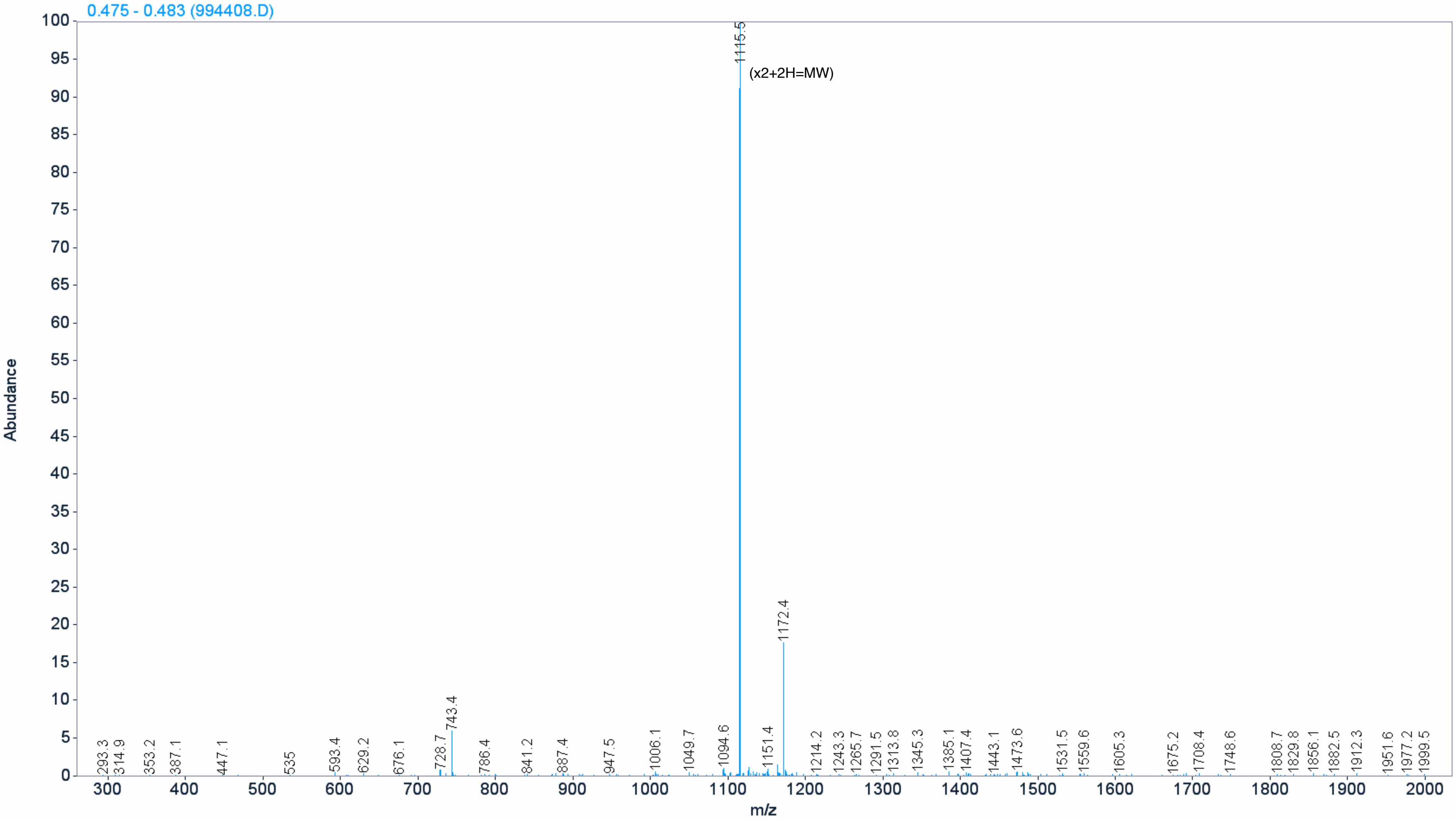

QC files for MP-793

CHINESE PEPTIDE

Certificate of Analysis

**Product Name:**	Acetyl-dPMI(5-12 alkyne staple, K9Q, 6xdAla tail)	**Barcode#:**	0955845343 0154692830
**Product #:**	991274	**Lot #:**	CT-12-01579

Sequence: Ac-DThr-DAla-DTrp(6-F)-DTyr-(S)-2-amino-2-methylhept-6-ynoic acid-DAsn-DPhe(4-CF3)-DGlu-DGln-DLeu-DLeu-(R)-2-amino-2-methyloct-7-ynoic acid-DAla-DAla-DAla-DAla-DAla-DAla-NH2(oalkyne coupling between (R)-2-amino-2-methyloct-7-ynoic acid and (S)-2-amino-2-methylhept-6-ynoic acid)

Molecular Weight: 2124.3

Mass Spectral Analysis: Electrospray "Exhibits correct MW" (see attached MS spectrogram)

HPLC Analysis: Peptide purity: 94.2% (see attached RP-HPLC chromatogram)

Solubility: 1 mg/ml in DMSO

Appearance: White lyophilized powder

Counter Ion: Ammonium salt

Cert. of Analysis Remarks:

Remarks: **Not for human use, research purpose only.**

Quality Assurance By: Hawk Zhu **Date: 03/09/21**
Quality Control Department

Chinese Peptide Company
No.69,12 Street,Hangzhou Economic & Technological Development Zone,China 310018
Tel: 86-571-86737011 86737118-8686
Fax: 86-571-86737013
Email: sales@chinesepeptide.com
Website: www.chinesepeptide.com

Data file:C:\HPCHEM\1\DATA\030821F\991274F.D   Sample Name:FINAL
Sample ID:991274   Lot:CT-12-01579
Mobile phase:A:0.1% TFA in H2O
B:0.09% TFA in (80%ACN+20%H2O)
Flow:1.0 ml/min   54.0%-64.0% B buffer in 20 min
Column:Phenomenex C18(2) 5u 100A 4.6*150mm A1112#

QC files for MP-793

===>

Injection Date : 3/8/2021   Location : Vial 61
Sample Name : Final   Inj. Vol. : 10 µl
Acq Operator : ZWJ
Acq. Method : Final.M
Analysis Method : C:\HPCHEM\1\METHODS\FINAL.M

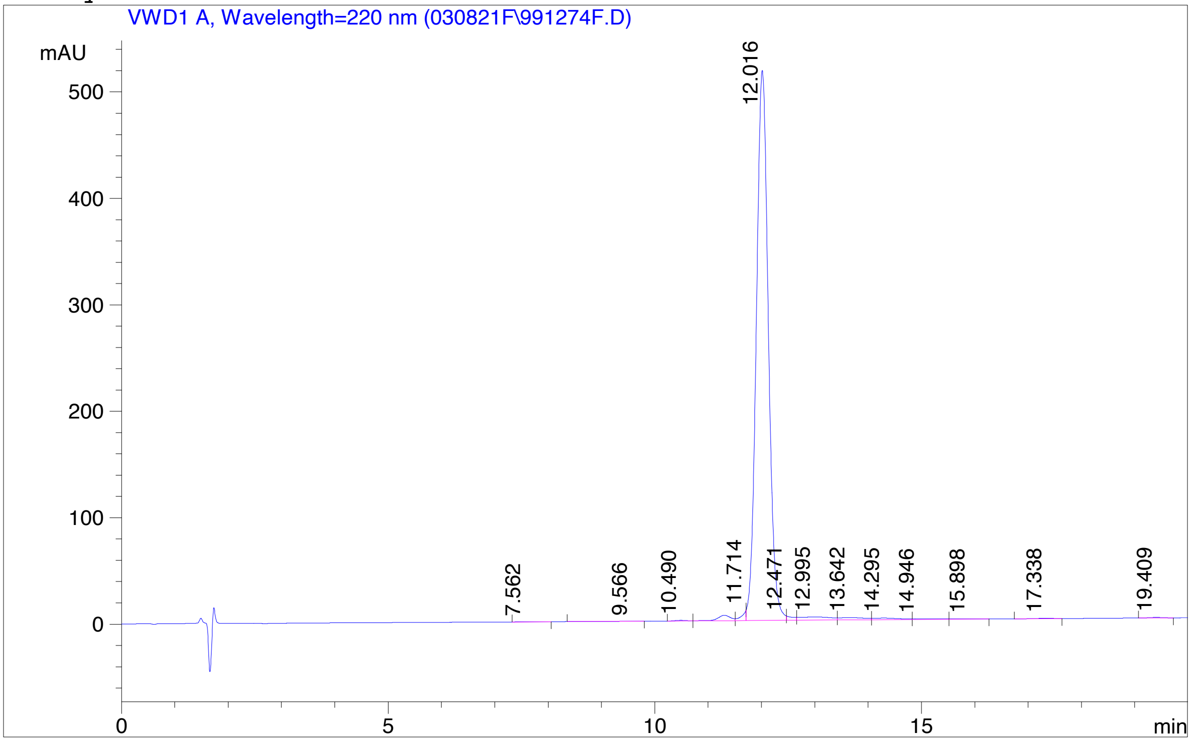

===>

Signal 1:VWD1 A, Wavelength=220 nm

Peak #	RT [min]	Type	Width [min]	Height	Area	Area %
1	7.562	MM	0.280	0.193	3.242	0.038
2	9.566	MM	0.728	0.169	7.401	0.086
3	10.490	BV	0.237	0.680	10.436	0.122
4	11.306	VV	0.255	5.136	86.728	1.013
5	11.714	VV	0.088	10.049	52.796	0.617
6	12.016	VF	0.241	515.641	8068.768	94.270
7	12.471	VV	0.144	4.066	35.154	0.411
8	12.995	VV	0.595	3.022	122.490	1.431
9	13.642	VV	0.455	2.346	75.005	0.876
10	14.295	VV	0.479	1.575	52.057	0.608
11	14.946	VV	0.446	0.529	17.055	0.199
12	15.898	VV	0.476	0.264	9.566	0.112
13	17.338	VV	0.377	0.350	8.518	0.100
14	19.409	VP	0.297	0.527	10.010	0.117

*** End of Report ***

QC files for MP-793

Data file: D:\DATE\210308\20210308-3 2021-03-08 14-02-21\991274F.D 3/8/2021 2:17:02 PM
Sample name: 991274
Description: Lot#:CT-12-01579
MW:2124.3
Sample Type:Final
Acq. method: 290-2000-Negative-
Frag 130v.M

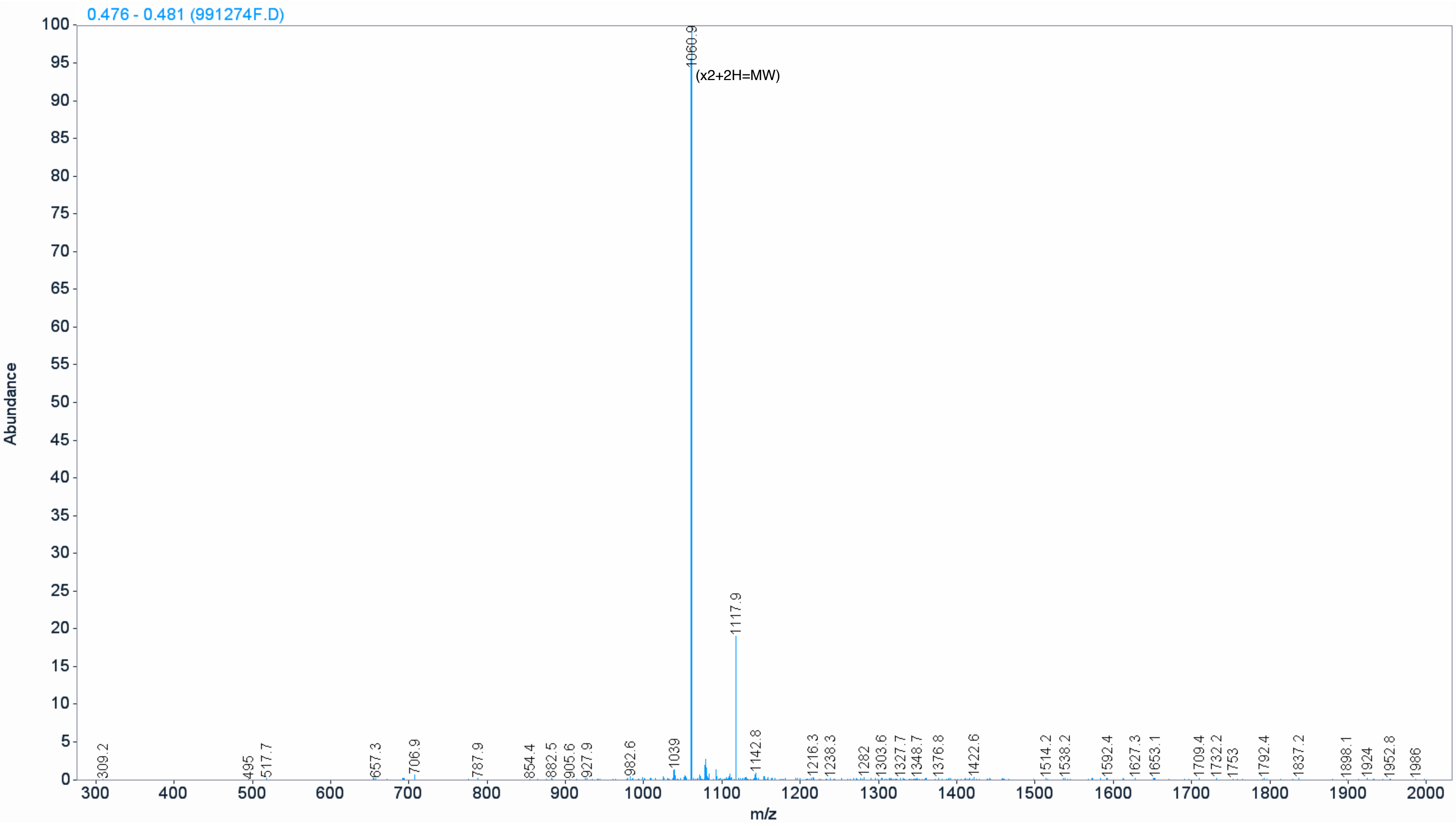

QC files for MP−616

CHINESE PEPTIDE

Certificate of Analysis

**Product Name:**	azide-ATSP-7041(alkyne staple, AAAAAa tail)	**Barcode#:**	0955842052 0154692762
**Product #:**	986286	**Lot #:**	CT-11-00100

Sequence: Ac-Lys(N3)-BetaAla-Leu-Thr-Phe-((R)-2-amino-2-methyloct-7-ynoic acid)-Glu-Tyr-Trp-Ala-Gln-Cba-((S)-2-amino-2-methylhept-6-ynoic acid)-Ala-Ala-Ala-Ala-Ala-D_Ala-NH2 (alkyne coupling between (R)-2-amino-2-methyloct-7-ynoic acid and (S)-2-amino-2-methylhept-6-ynoic acid)

Molecular Weight: 2161.5

Mass Spectral Analysis: Electrospray “Exhibits correct MW” (see attached MS spectrogram)

HPLC Analysis: Peptide purity: 95.1% (see attached RP-HPLC chromatogram)

Solubility: 1 mg/ml in DMSO

Appearance: White lyophilized powder

Counter Ion: No Salt

Cert. of Analysis Remarks:

Remarks: **Not for human use, research purpose only.**

Quality Assurance By: Hawk Zhu **Date: 02/19/21**

Quality Control Department

Chinese Peptide Company
No.69,12 Street,Hangzhou Economic & Technological Development Zone,China 310018
Tel: 86-571-86737011 86737118-8686
Fax: 86-571-86737013
Email: sales@chinesepeptide.com
Website: www.chinesepeptide.com

Data file:C:\HPCHEM\1\DATA\021921F\986286F.D Sample Name:FINAL
Sample ID:986286 Lot:CT-11-00100
Mobile phase:A:0.1% TFA in H2O
B:0.09% TFA in (80%ACN+20%H2O)
Flow:1.0 ml/min 70.0%-80.0% B buffer in 20 min
Column:Phenomenex C18(2) 5u 100A 4.6*150mm A1112#

QC files for MP-616

==>

Injection Date : 2/19/2021 Location : Vial 32
Sample Name : Final Inj. Vol. : 7 µl
Acq Operator : ZWJ
Acq. Method : Final.M
Analysis Method : C:\HPCHEM\1\METHODS\FINAL.M

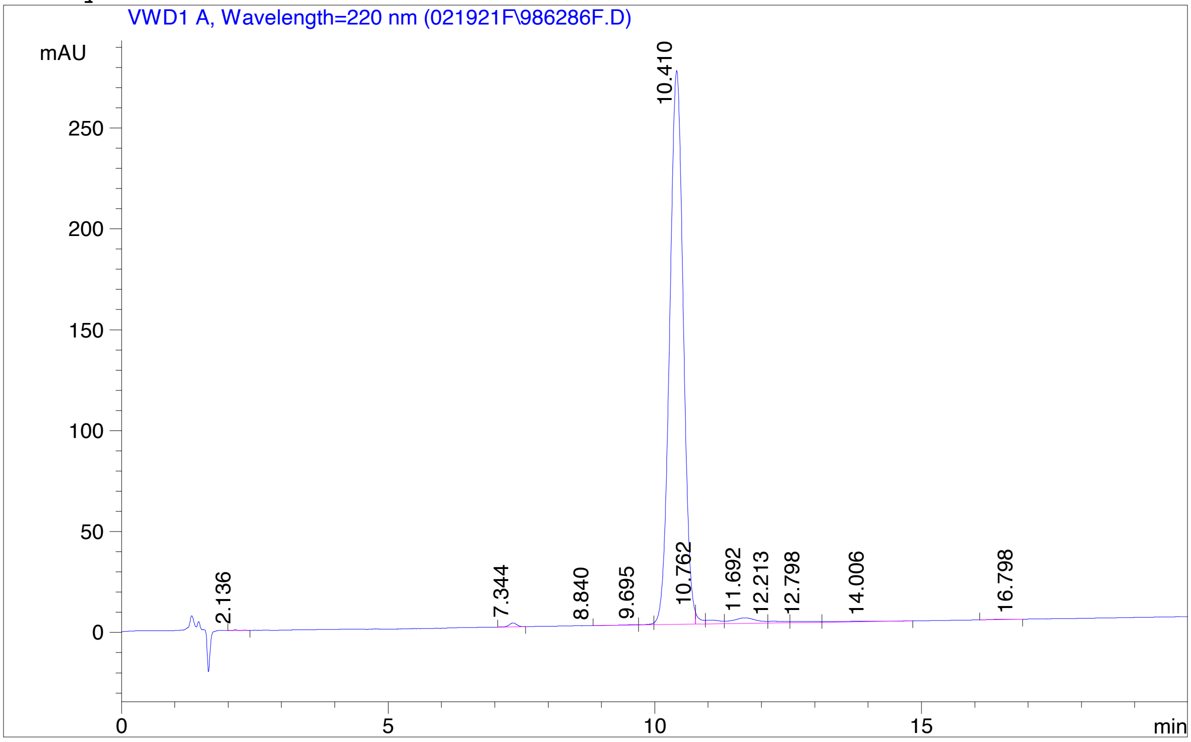

==>

Signal 1:VWD1 A, Wavelength=220 nm

Peak #	RT [min]	Type	Width [min]	Height	Area	Area %
1	2.136	MM	0.168	0.342	3.436	0.067
2	7.344	VV	0.162	1.973	20.430	0.400
3	8.840	VV	2.985	0.030	5.389	0.106
4	9.695	VV	0.576	0.160	5.545	0.109
5	10.410	VF	0.277	274.189	4859.660	95.143
6	10.762	VV	0.087	6.073	31.673	0.620
7	11.058	VV	0.265	1.916	35.790	0.701
8	11.692	VV	0.452	2.800	87.456	1.712
9	12.213	VV	0.305	0.867	19.211	0.376
10	12.798	VV	0.455	0.546	18.036	0.353
11	14.006	VV	1.281	0.162	17.435	0.341
12	16.798	MM	1.552	0.039	3.674	0.072

*** End of Report ***

QC files for MP-616

Data file: D:\DATE\210219\20210219-1 2021-02-19 10-00-20\986286F.D 2/19/2021 12:48:02 PM
Sample name: 986286
Description: Lot#:CT-11-00100
MW:2161.5
Sample Type:Final
Acq. method: 290-2000-Negative-
Frag 130v.M

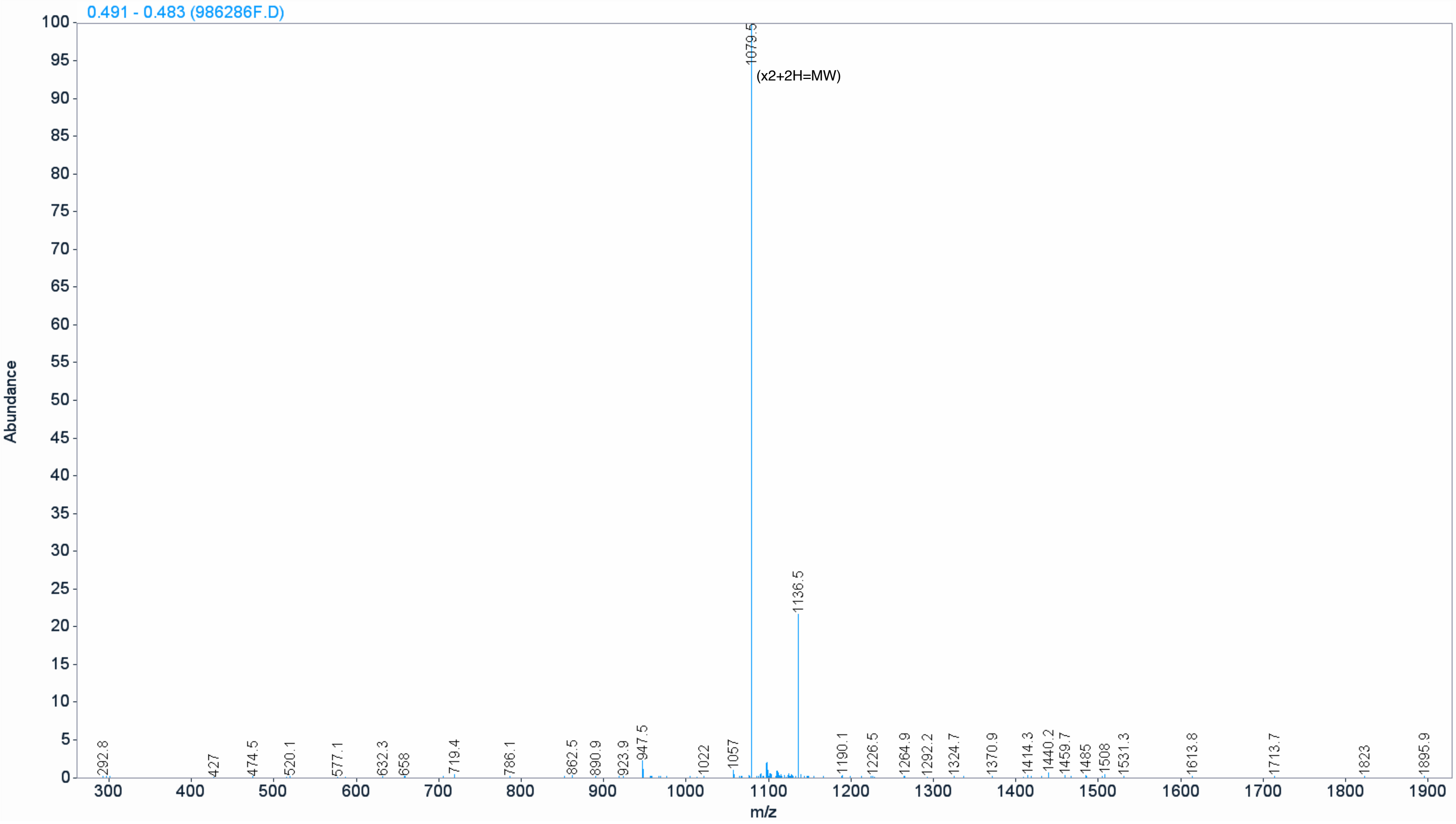

QC files for MP-4897 (ALRN-6924)

CHINESE PEPTIDE

Certificate of Analysis

**Product Name:**	putative-6924	**Barcode#:**	0955841567<br>0154691598
**Product #:**	991264	**Lot #:**	CT-12-01589
**Sequence:**	Ac-Leu-Thr-Phe-R8-Glu-Tyr-Trp-Ala-Gln-Leu-S5-Ala-Ala<br>-Ala-Ala-Ala-DAla-NH2 (olefin staple from R8 to S5)		
**Molecular Weight:**	1930.3		
**Mass Spectral Analysis:**	Electrospray "Exhibits correct MW"<br>(see attached MS spectrogram)		
**HPLC Analysis:**	Peptide purity: 97.8%<br>(cis/trans isomers mixture see attached RP-HPLC chromatogram)		
**Solubility:**	1 mg/ml in DMSO		
**Appearance:**	White lyophilized powder		
**Counter Ion:**	Ammonium salt		
**Cert. of Analysis Remarks:**			

Remarks: **Not for human use, research purpose only.**

Quality Assurance By: Hawk Zhu **Date: 01/19/21**

Quality Control Department

Chinese Peptide Company
No.69,12 Street,Hangzhou Economic & Technological Development Zone,China 310018
Tel: 86-571-86737011 86737118-8686
Fax: 86-571-86737013
Email: sales@chinesepeptide.com
Website: www.chinesepeptide.com

Data file:C:\HPCHEM\1\DATA\011821F\991264F.D   Sample Name:FINAL
Sample ID:991264   Lot:CT-12-01589
Mobile phase:A:0.1% TFA in H2O
B:0.09% TFA in (80%ACN+20%H2O)
Flow:1.0 ml/min   82.0%-92.0% B buffer in 20 min
Column:Phenomenex C18(2) 5u 100A 4.6*150mm A1112#

QC files for MP-4897 (ALRN-6924)

===->

Injection Date : 1/18/2021   Location : Vial 3
Sample Name : Final   Inj. Vol. : 10 µl
Acq Operator : ZWJ
Acq. Method : Final.M
Analysis Method : C:\HPCHEM\1\METHODS\FINAL.M

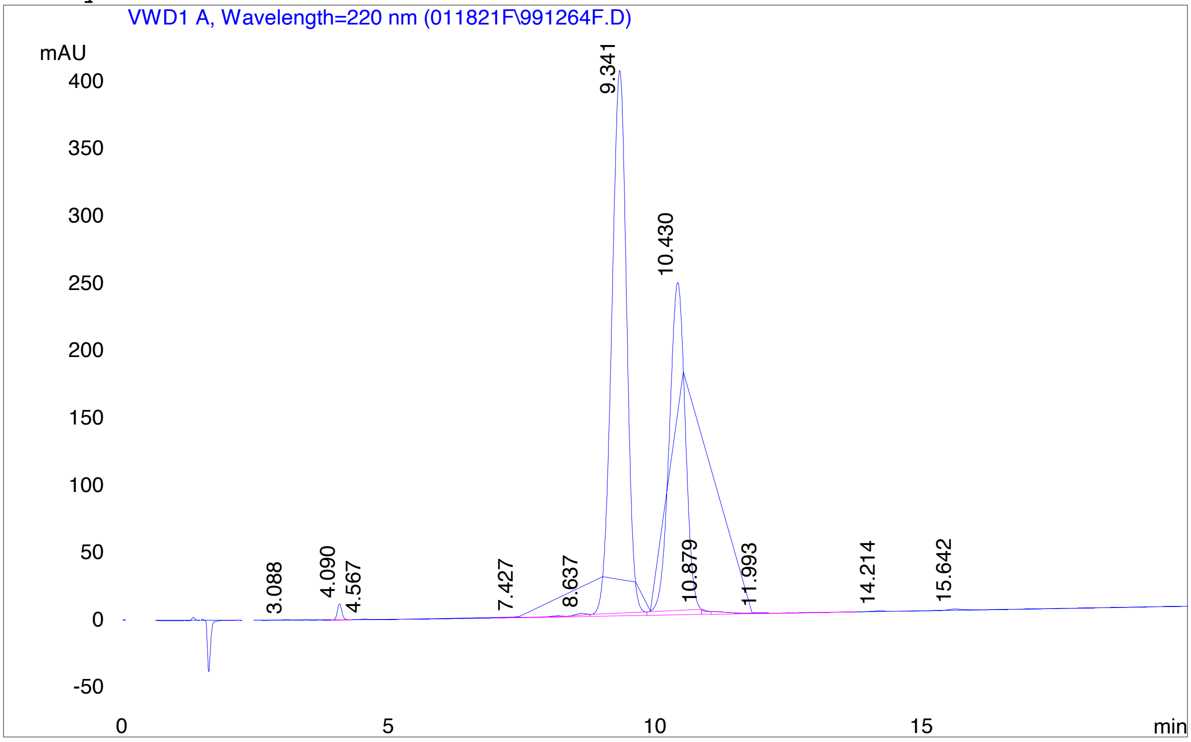

===->

Signal 1:VWD1 A, Wavelength=220 nm

Peak #	RT [min]	Type	Width [min]	Height	Area	Area %
1	3.088	MM	0.454	0.298	8.105	0.060
2	4.090	VV	0.099	11.928	71.092	0.526
3	4.567	VP	0.158	0.551	5.527	0.041
4	7.427	VV	0.239	0.449	7.156	0.053
5	8.183	VV	0.296	0.886	17.648	0.131
6	8.637	VV	0.272	2.340	41.712	0.309
7	9.341	VV	0.311	404.993	7968.135	58.992
8	10.430	VF	0.330	246.875	5249.694	38.866
9	10.879	VF	0.118	3.806	26.946	0.199
10	11.053	VV	0.375	2.140	48.158	0.357
11	11.993	VV	0.426	0.614	18.754	0.139
12	13.791	VV	2.450	0.099	14.518	0.107
13	14.214	VV	0.283	0.616	11.556	0.086
14	15.642	VV	0.280	1.008	18.158	0.134

===

*** End of Report ***

QC files for MP-4897 (ALRN-6924)

Data file: D:\DATE\210118\20210118-1 2021-01-18 11-19-51\991264.D 1/18/2021 3:35:59 PM
Sample name: 991264
Description: Lot#:CT-12-01589
MW:1930.3
Sample Type:Final
Acq. method: 290-2000-Negative-
Frag 130v.M

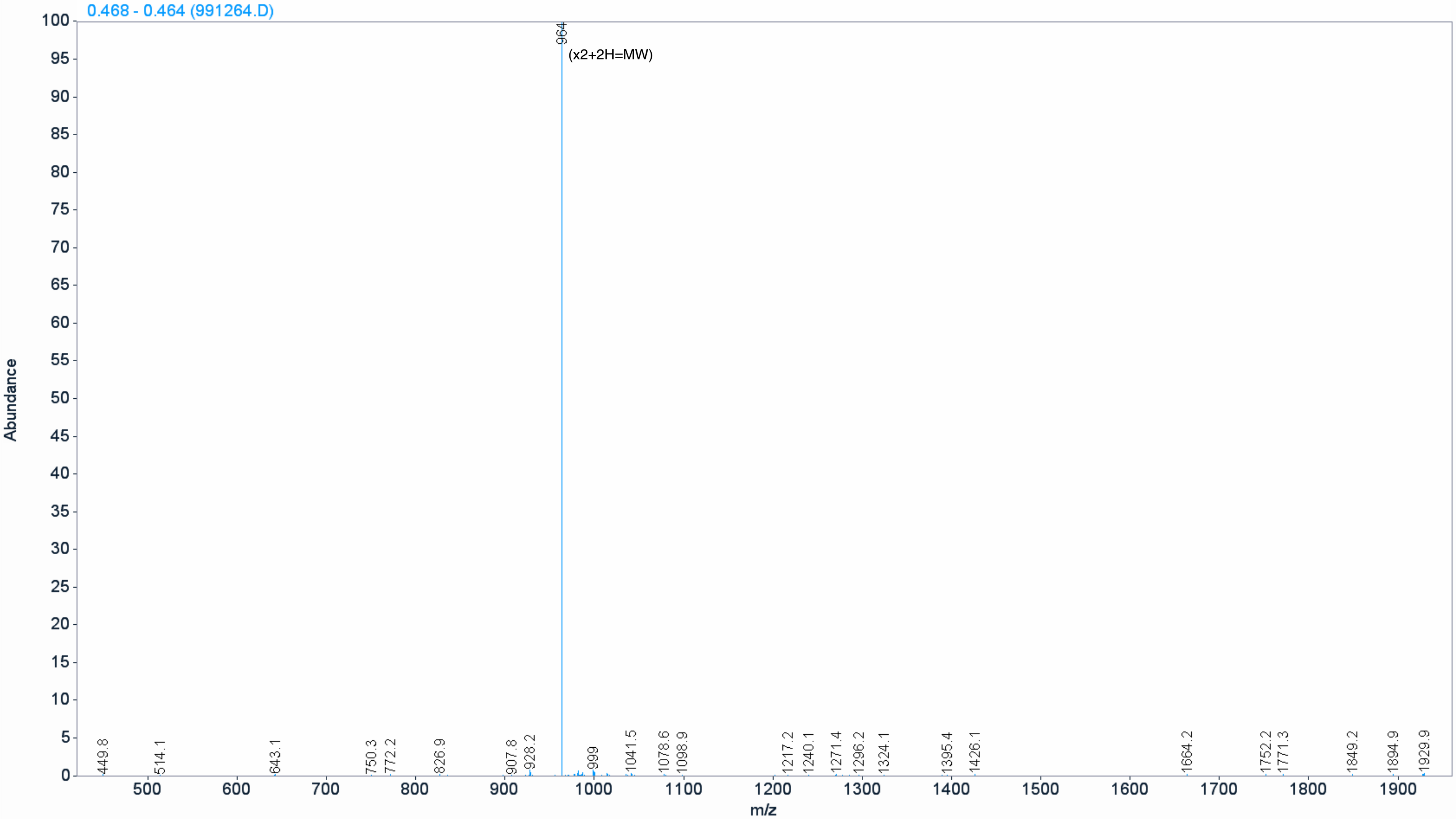

QC files for MP-042

CHINESE PEPTIDE

Certificate of Analysis

**Product Name:**	DPMI (6-10) (K9Q, R12S, dAdAdAdAdAdA tail)	**Barcode#:**	0953968464
**Product #:**	988999	**Lot #:**	CT-08-00054
**Sequence:**	Ac-DThr-DAla-DTrp(6-F)-DTyr-DAla-R5-DPhe(4-CF3)-DGlu-DGln-R5-DLeu-DSer-DAla-DAla-DAla-DAla-DAla-DAla-NH2(olefin staple from R5 to R5)		
**Molecular Weight:**	2019.2		
**Mass Spectral Analysis:**	Electrospray “Exhibits correct MW” (see attached MS spectrogram)		
**HPLC Analysis:**	Peptide purity: 90.2% (see attached RP-HPLC chromatogram)		
**Solubility:**	1 mg/ml in DMSO		
**Appearance:**	White lyophilized powder		
**Counter Ion:**	No Salt		
**Cert. of Analysis Remarks:**			

Remarks: **Not for human use, research purpose only.**

Quality Assurance By: Hawk Zhu **Date: 09/09/20**
Quality Control Department

Chinese Peptide Company
No.69,12 Street,Hangzhou Economic & Technological Development Zone,China 310018
Tel: 86-571-86737011 86737118-8686
Fax: 86-571-86737013
Email: sales@chinesepeptide.com
Website: www.chinesepeptide.com

Data file:C:\HPCHEM\1\DATA\090720F\988999F.D   Sample Name:FINAL
Sample ID:988999   Lot:CT-08-00054
Mobile phase:A:0.1% TFA in H2O
B:0.09% TFA in (80%ACN+20%H2O)
Flow:1.0 ml/min   62.0%-72.0% B buffer in 20 min
Column:Phenomenex C18(2) 5u 100A 4.6*150mm A1112#

QC files for MP-042

===-->

Injection Date : 9/7/2020 Location : Vial 34
Sample Name : Final Inj. Vol. : 10 µl
Acq Operator : ZWJ
Acq. Method : Final.M
Analysis Method : C:\HPCHEM\1\METHODS\FINAL.M

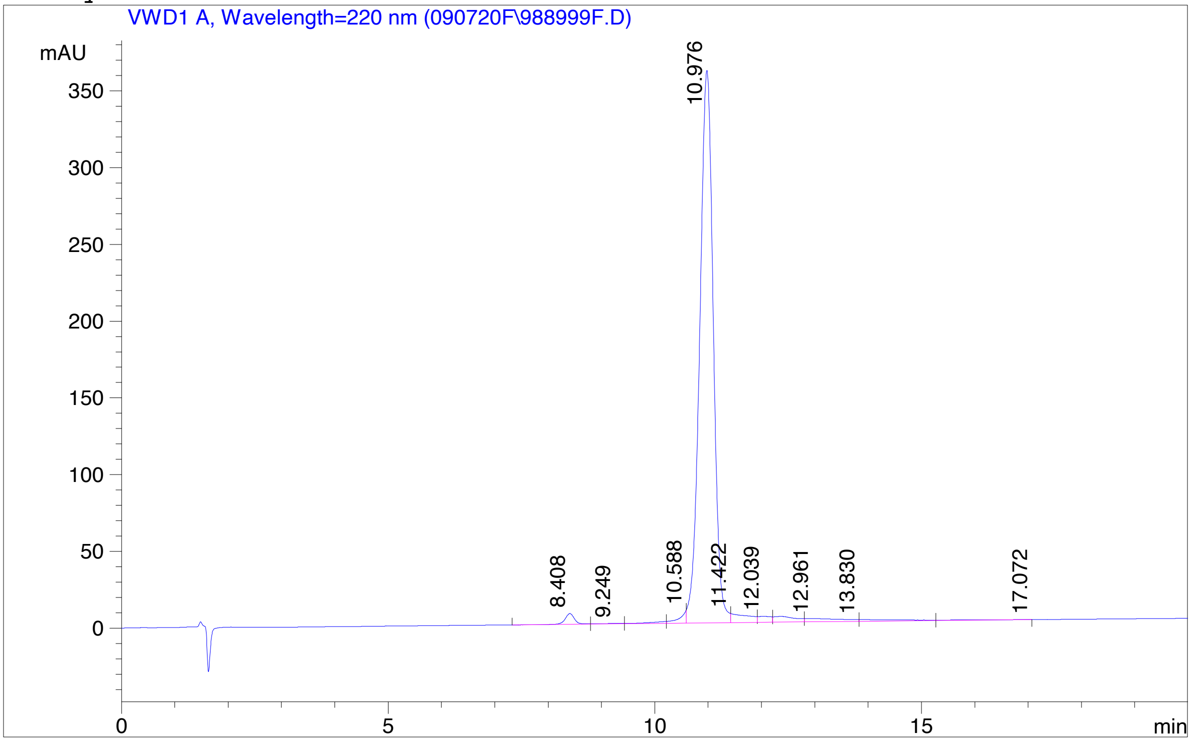

===-->

Signal 1:VWD1 A, Wavelength=220 nm

Peak #	RT [min]	Type	Width [min]	Height	Area	Area %
1	8.408	VV	0.194	7.019	88.786	1.260
2	9.249	VV	0.367	0.366	9.870	0.140
3	10.219	VV	0.359	1.268	27.277	0.387
4	10.588	VV	0.133	8.425	67.304	0.955
5	10.976	VF	0.276	359.441	6358.549	90.248
6	11.422	VV	0.387	5.992	139.228	1.976
7	12.039	VV	0.236	3.896	65.221	0.926
8	12.361	VV	0.401	3.769	107.007	1.519
9	12.961	VF	0.651	2.124	103.732	1.472
10	13.830	VV	0.872	1.185	61.984	0.880
11	17.072	VV	25.829	0.011	16.691	0.237

===

*** End of Report ***

QC files for MP-042

Data file: D:\DATE\200907\20200907-1 2020-09-07 10-34-41\988999.D 9/7/2020 11:42:39 AM

Sample name: 988999

Description: Lot#:CT-08-00054
MW:2019.2
Sample Type:Final

Acq. method: 290-2000-Negative-
Frag 130v.M

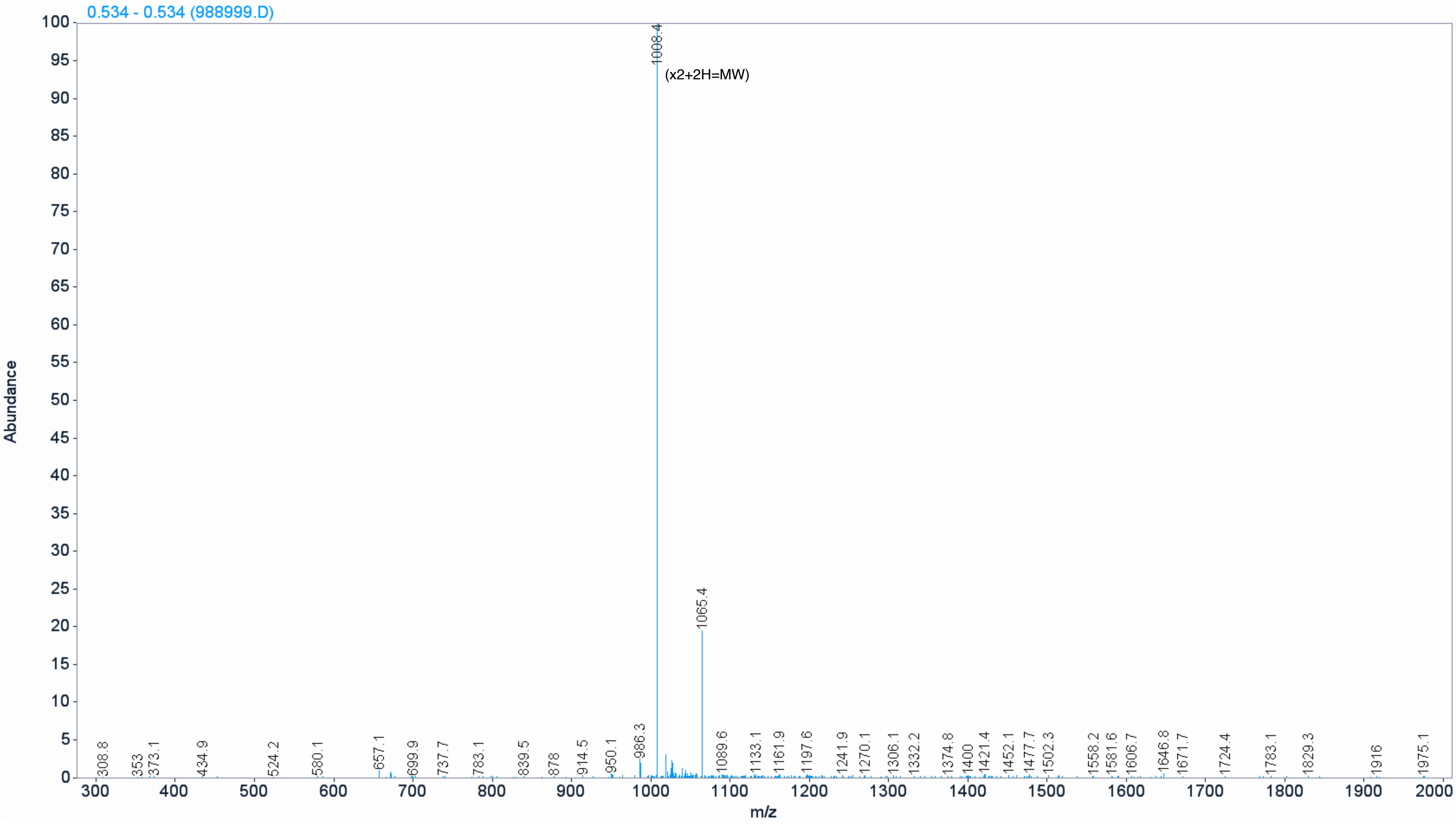

QC files for MP-797

CHINESE PEPTIDE

Certificate of Analysis

**Product Name:**	Acetyl-DPMI (6-10) (K9Q, R12S)	**Barcode#:**	0955004740 0154300124
**Product #:**	987308	**Lot #:**	CT-04-00926

Sequence: Ac-DThr-DAla-DTrp(6-F)-DTyr-DAla-R5-DPhe(4-CF3)-DGlu-DGln-R5-DLeu-DSer-NH2 (olefin staple from R5 to R5)

Molecular Weight: 1592.7

Mass Spectral Analysis: Electrospray “Exhibits correct MW” (see attached MS spectrogram)

HPLC Analysis: Peptide purity: 91.8% (see attached RP-HPLC chromatogram)

Solubility: 1 mg/ml in DMSO

Appearance: Off-white lyophilized powder

Counter Ion: No Salt

Cert. of Analysis Remarks:

Remarks: **Not for human use, research purpose only.**

Quality Assurance By: Hawk Zhu **Date: 05/07/20**
Quality Control Department

Chinese Peptide Company
No.69,12 Street,Hangzhou Economic & Technological Development Zone,China 310018
Tel: 86-571-86737011 86737118-8686
Fax: 86-571-86737013
Email: sales@chinesepeptide.com
Website: www.chinesepeptide.com

Data file:C:\HPCHEM\1\DATA\050420\987308F.D   Sample Name:FINAL
Sample ID:987308   Lot:CT-04-00926
Mobile phase:A:0.1% TFA in H2O
B:0.09% TFA in (80%ACN+20%H2O)
Flow:1.0 ml/min   56.0%-66.0% B buffer in 20min
Column:Phenomenex C18(2) 5u 100A 4.6*150mm A1112#

QC files for MP-797

===->

Injection Date : 5/4/2020   Location : Vial 71
Sample Name : Final   Inj. Vol. : 10 µl
Acq Operator : ZWJ
Acq. Method : Final.M
Analysis Method : C:\HPCHEM\1\METHODS\FINAL.M

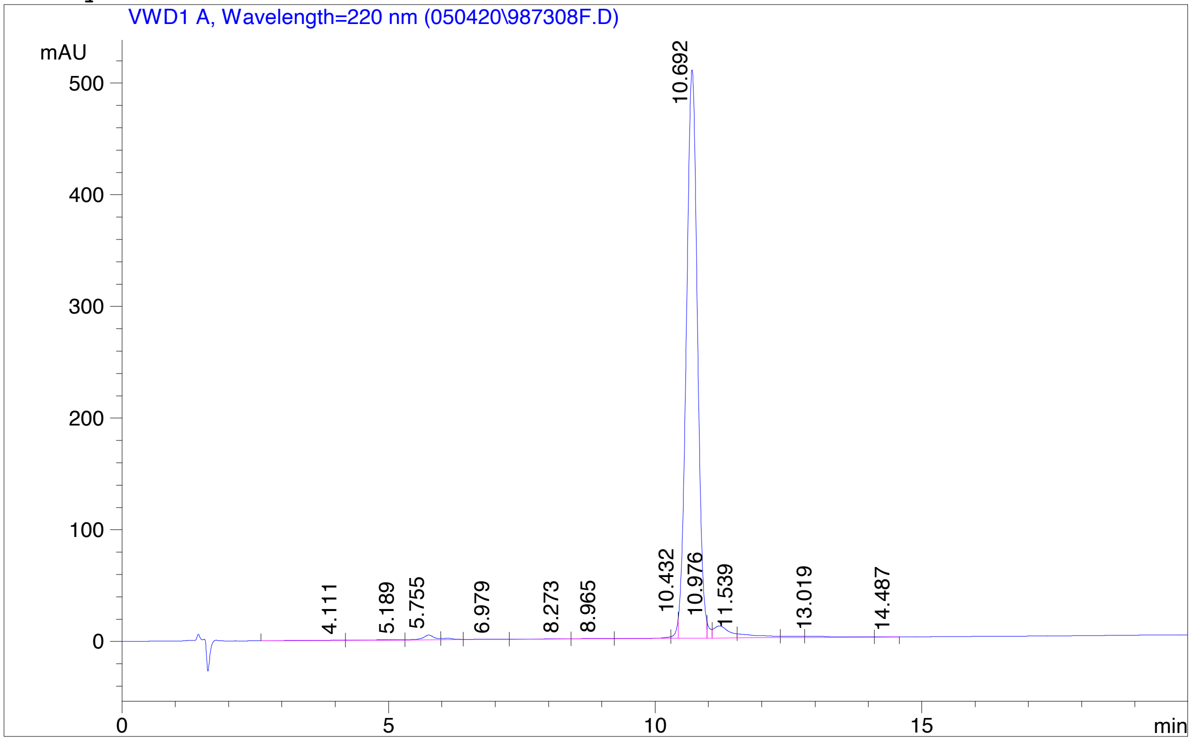

===->

Signal 1:VWD1 A, Wavelength=220 nm

Peak #	RT [min]	Type	Width [min]	Height	Area	Area %
1	4.111	VV	0.786	0.213	13.499	0.169
2	5.189	VV	0.650	0.267	14.203	0.178
3	5.755	VV	0.212	4.280	62.723	0.788
4	6.099	VV	0.216	1.316	19.777	0.248
5	6.979	VV	0.459	0.351	12.235	0.154
6	8.273	VV	0.514	0.195	7.832	0.098
7	8.965	VV	0.453	0.287	10.000	0.126
8	10.296	VV	0.159	1.463	14.001	0.176
9	10.432	VV	0.044	17.260	45.820	0.575
10	10.692	VF	0.226	507.441	7318.391	91.892
11	10.976	VV	0.063	14.148	53.316	0.669
12	11.207	VF	0.280	10.869	211.797	2.659
13	11.539	VV	0.473	3.646	103.571	1.300
14	12.579	VV	0.359	1.311	33.187	0.417
15	13.019	VV	0.482	1.192	41.510	0.521
16	14.487	VV	0.474	0.066	2.236	0.028

===

*** End of Report ***

QC files for MP-797

Qualitative Analysis Report

**Data Filename**	987308F1.d	**Sample Name**	987308
**Sample Type**	Sample	**Position**	P1-F5
**Instrument Name**	6530	**User Name**	SCSCOE6530\justin.jia
**Acq Method**	ms1-300-negative.m	**Acquired Time**	5/4/2020 2:44:24 PM
**IRM Calibration Status**	Success	**DA Method**	BioConfirmSyntheticPeptide-Default.m
**Comment**	Instrument ID:2013110801		

Sample Group **Info.** Sample:987308 Code:N/A Lot#:CT-04-00926

Acquisition SW Version 6200 series TOF/6500 series Q-TOF B.05.01 (B5125.1)

User Spectra

Fragmentor Voltage	Collision Energy	Ionization Mode
135	0	ESI

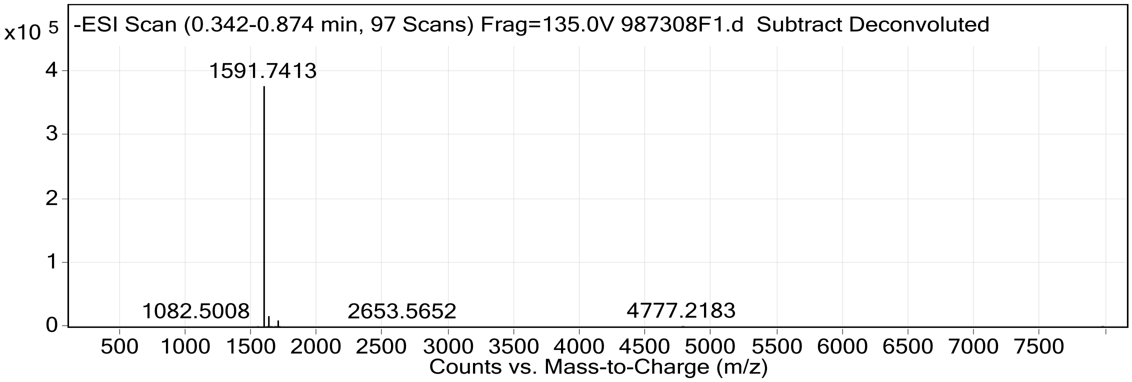

Peak List

m/z	Abund
1591.7413	378216.16
1592.7443	345325.19
1593.7464	166353.91
1594.7478	56887.68
1595.7486	12895.7
1596.7483	2930.2
1627.7163	17661.71
1628.7188	16061.65
1629.7163	14607.41
1630.7161	8851.77
1631.717	3996.8
1689.7068	2919.63
1705.7442	11330.66
1706.7411	10110.76
1707.7409	5045.28

--- End Of Report ---

QC files for MP-202

CHINESE PEPTIDE

Certificate of Analysis

**Product Name:**	azido-ATSP7041<br>(F3 to dPhe)	**Barcode#:**	0954308047<br>0153962811
**Product #:**	981783	**Lot #:**	CS-03-00296
**Sequence:**	Ac-Lys(N3)-betaAla-Leu-Thr-DPhe-R8-Glu-Tyr-Trp-Ala-Gln<br>-Cba-S5-Ser-Ala-Ala-NH2(olefin staple from R8 to S5)		
**Molecular Weight:**	1970.3		
**Mass Spectral Analysis:**	Electrospray “Exhibits correct MW”<br>(see attached MS spectrogram)		
**HPLC Analysis:**	Peptide purity: 95.2%<br>(cis/trans isomers mixture see attached RP-HPLC chromatogram)		
**Solubility:**	1 mg/ml in DMSO		
**Appearance:**	White lyophilized powder		
**Counter Ion:**	No Salt		
**Cert. of Analysis Remarks:**			

Remarks: **Not for human use, research purpose only.**

Quality Assurance By: Hawk Zhu **Date: 03/21/19**
Quality Control Department

Chinese Peptide Company
No.69,12 Street,Hangzhou Economic & Technological Development Zone,China 310018
Tel: 86-571-86737011 86737118-8686
Fax: 86-571-86737013
Email: sales@chinesepeptide.com
Website: www.chinesepeptide.com

```
Data file:C:\HPCHEM\1\DATA\032119\981783F.D      Sample Name:FINAL
Sample ID:981783        Lot:CS-03-00296
Mobile phase:A:0.1% TFA in H2O
             B:0.09% TFA in (80%ACN+20%H2O)
Flow:1.0 ml/min     65.0%-75.0% B buffer in 20min
Column:Phenomenex C18(2) 5u 100A 4.6*150mm A1112#
```

QC files for MP-202

```
=========================================================================->
Injection Date  : 3/21/2019                        Location   :    Vial 22
Sample Name     : Final                            Inj. Vol.  :     10 µl
Acq Operator    : ZWJ
Acq. Method     : Final.M
Analysis Method : C:\HPCHEM\1\METHODS\FINAL.M
```

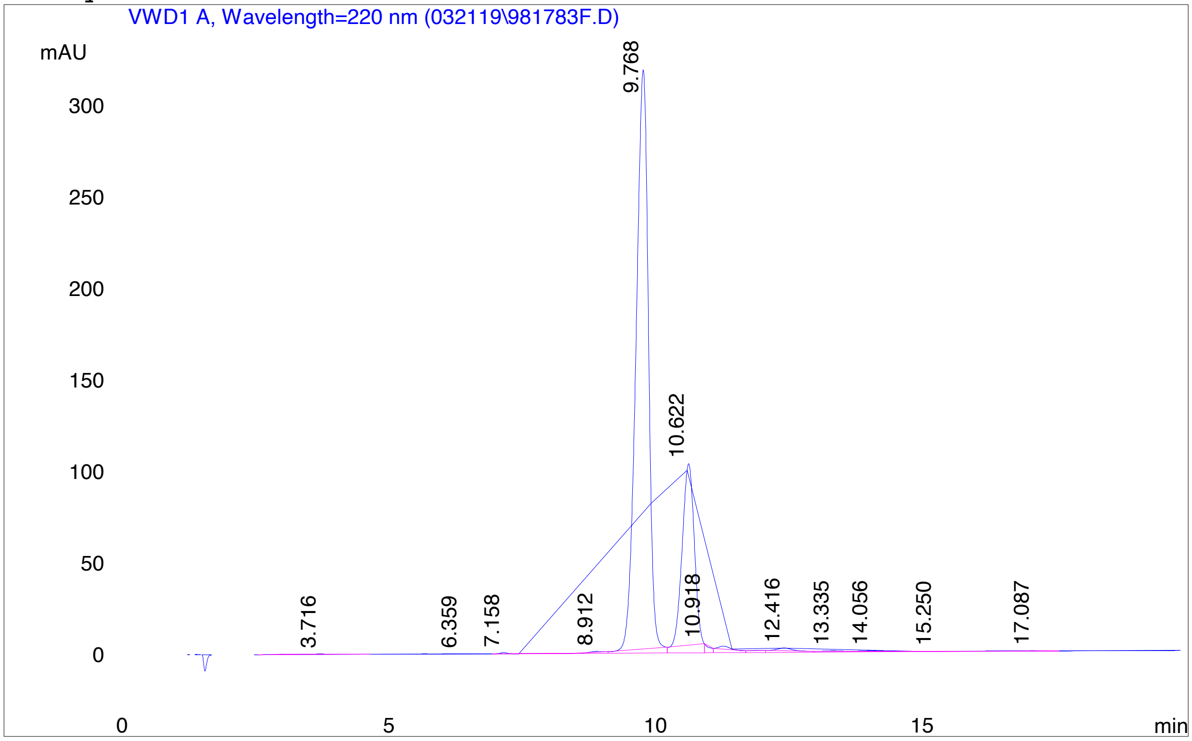


```
=========================================================================->
```

Signal 1:VWD1 A, Wavelength=220 nm

Peak #	RT [min]	Type	Width [min]	Height	Area	Area %
1	3.254	VV	0.398	0.256	7.579	0.104
2	3.716	VV	0.526	0.303	12.305	0.169
3	6.359	MM	1.140	0.031	2.105	0.029
4	6.835	VV	0.205	0.092	1.200	0.016
5	7.158	VV	0.152	0.642	6.107	0.084
6	8.491	VV	0.696	0.047	1.956	0.027
7	8.912	VV	0.335	1.023	22.193	0.305
8	9.768	VV	0.255	318.358	5212.101	71.624
9	10.622	VF	0.265	103.188	1721.343	23.654
10	10.918	VV	0.106	4.967	31.574	0.434
11	11.275	VV	0.333	3.565	82.208	1.130
12	11.951	VV	0.304	1.133	24.464	0.336
13	12.416	VV	0.436	2.313	71.401	0.981
14	13.335	VV	0.473	0.747	25.440	0.350
15	14.056	VV	0.568	0.660	26.230	0.360
16	15.250	VV	0.546	0.191	8.440	0.116
17	17.087	VV	1.349	0.181	20.423	0.281

*** End of Report ***

QC files for MP-202

**Data file:**	D:\DATE\190321\20190321-2 2019-03-21 13-02-59\981783.D	3/21/2019 1:22:50 PM
**Sample name:**	981783	
**Description:**	Lot#:CS-03-00296<br>MW:1970.3<br>Sample Type:Final	
**Acq. method:**	290-2000-Negative-<br>Frag 170v.M	

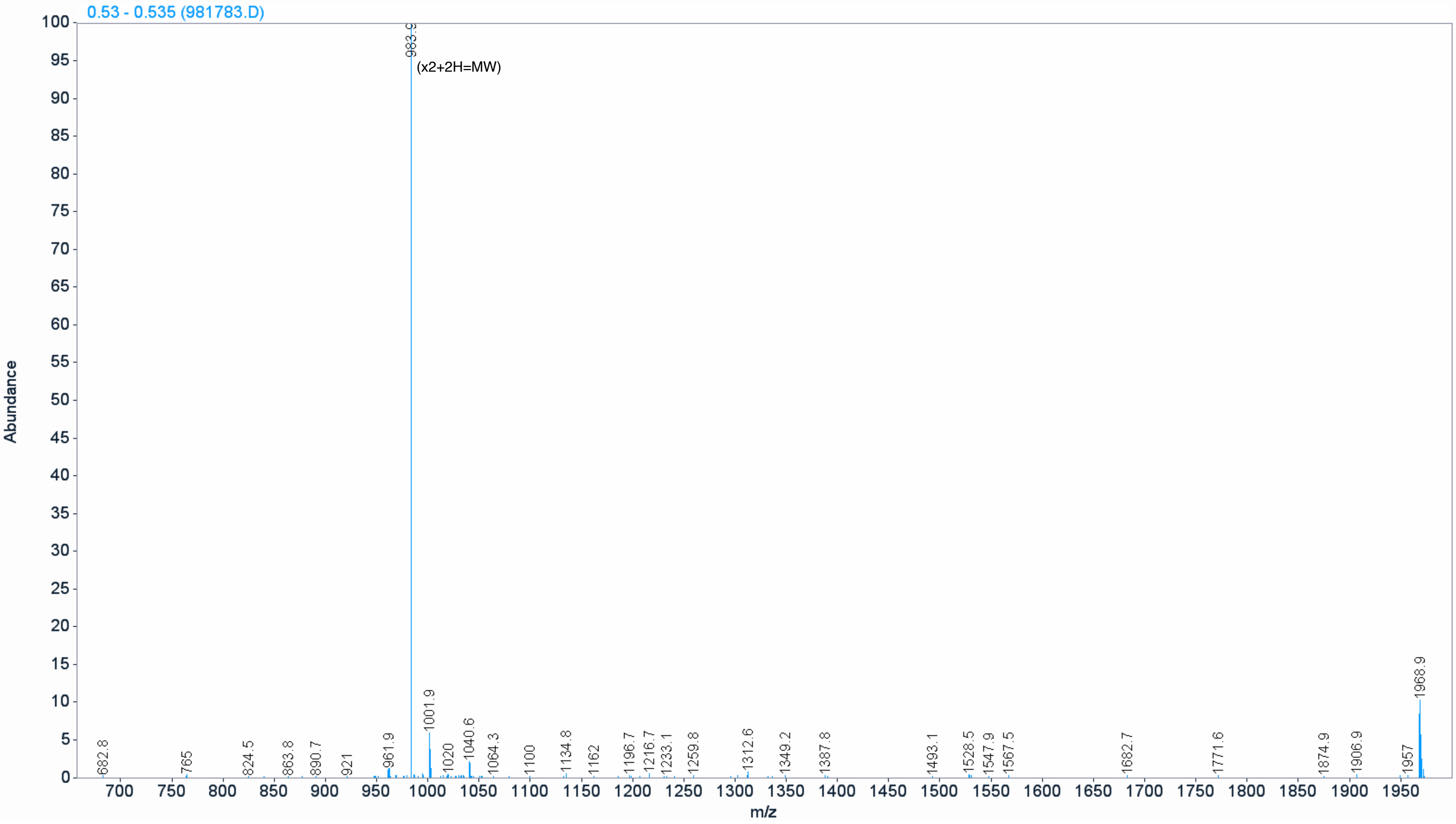

QC files for MP-081

CHINESE PEPTIDE

Certificate of Analysis

Product Name: Azide-ATSP-7041 (Mixed) **Barcode#:** 0953375668
0153943933

Product #: 970027 **Lot #:** CR-12-01058

Sequence: Ac-Lys(N3)-betaAla-Leu-Thr-Phe-R8-Glu-Tyr-Trp-Ala-Gln
-Cba-S5-Ser-Ala-Ala-NH2 (olefin staple from R8 to S5)

Molecular Weight: 1970.3

Mass Spectral Analysis: Electrospray "Exhibits correct MW"
(see attached MS spectrogram)

HPLC Analysis: Peptide purity: 95.6%
(cis/trans isomers mixture see attached RP-HPLC chromatogram)

Solubility: 1 mg/ml in DMSO

Appearance: White lyophilized powder

Counter Ion: Ammonium salt

Cert. of Analysis Remarks:

Remarks: **Not for human use, research purpose only.**

Quality Assurance By: Hawk Zhu **Date: 01/02/19**
Quality Control Department

Chinese Peptide Company
No.69,12 Street,Hangzhou Economic & Technological Development Zone,China 310018
Tel: 86-571-86737011 86737118-8686
Fax: 86-571-86737013
Email: sales@chinesepeptide.com
Website: www.chinesepeptide.com

Data file:C:\HPCHEM\1\DATA\122918\970027F.D Sample Name:FINAL
Sample ID:970027 Lot:CR-12-01058
Mobile phase:A:0.1% TFA in H2O
B:0.09% TFA in (80%ACN+20%H2O)
Flow:1.0 ml/min 74.0%-84.0% B buffer in 20 min
Column:Phenomenex C18(2) 5u 100A 4.6*150mm A1112#

QC files for MP-081

->
==->

Injection Date : 12/29/2018 Location : Vial 91
Sample Name : Final Inj. Vol. : 15 µl
Acq Operator : ZWJ
Acq. Method : Final.M
Analysis Method : C:\HPCHEM\1\METHODS\FINAL.M

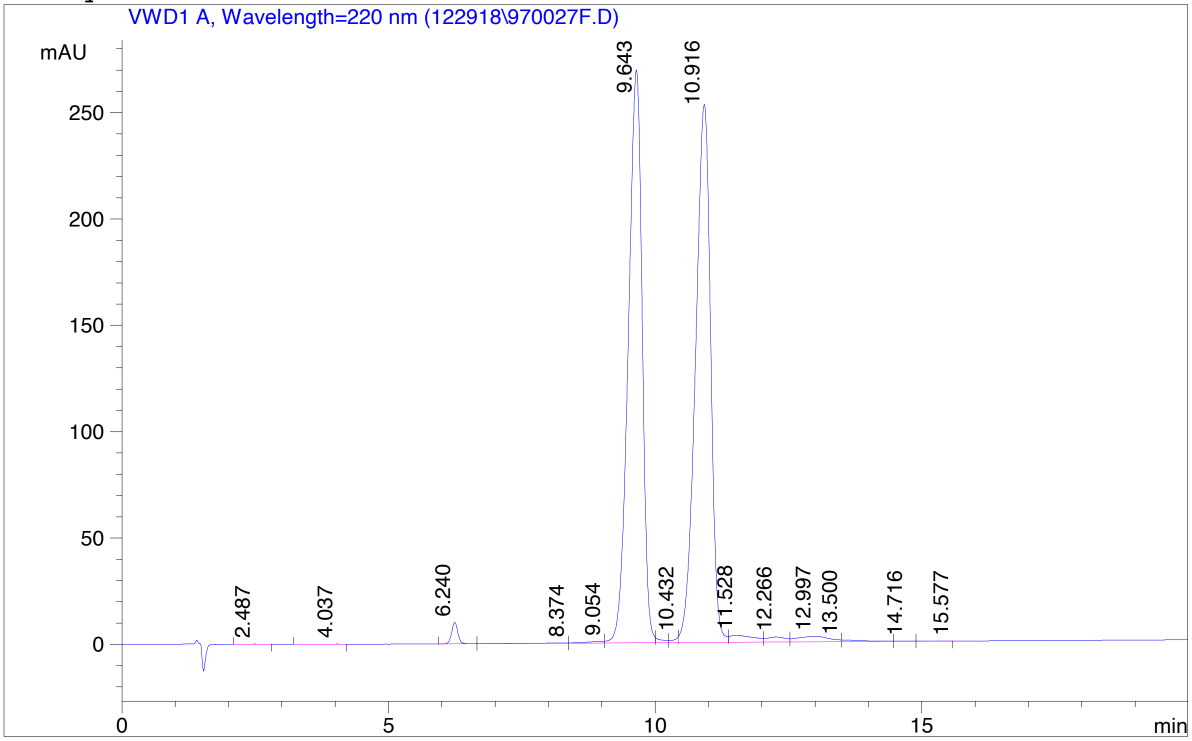

==->

Signal 1:VWD1 A, Wavelength=220 nm

Peak #	RT [min]	Type	Width [min]	Height	Area	Area %
1	2.487	MM	0.217	0.173	2.248	0.022
2	4.037	MM	0.518	0.084	2.617	0.026
3	6.240	VV	0.136	9.972	81.311	0.793
4	8.374	VV	0.728	0.112	4.914	0.048
5	9.054	VV	0.360	0.840	18.138	0.177
6	9.643	VF	0.287	268.419	4851.902	47.299
7	10.005	VV	0.139	2.407	20.031	0.195
8	10.432	VV	0.094	2.694	15.249	0.149
9	10.916	VV	0.311	252.755	4963.955	48.391
10	11.528	VV	0.425	3.319	102.563	1.000
11	12.266	VV	0.359	2.367	58.577	0.571
12	12.997	VF	0.584	2.643	109.860	1.071
13	13.500	VV	0.452	0.808	21.928	0.214
14	14.716	VV	0.356	0.110	2.759	0.027
15	15.577	VV	1.909	0.016	1.860	0.018

==

*** End of Report ***

QC files for MP-081

Data file: D:\DATE\181229\20181229-1 2018-12-29 10-44-55\970027F.D 12/29/2018 1:17:52 PM

Sample name: 970027

Description: Lot#:CR-12-01058
MW:1970.3
Sample Type:Final

Acq. method: 290-2000-Negative-
Frag 170v.M

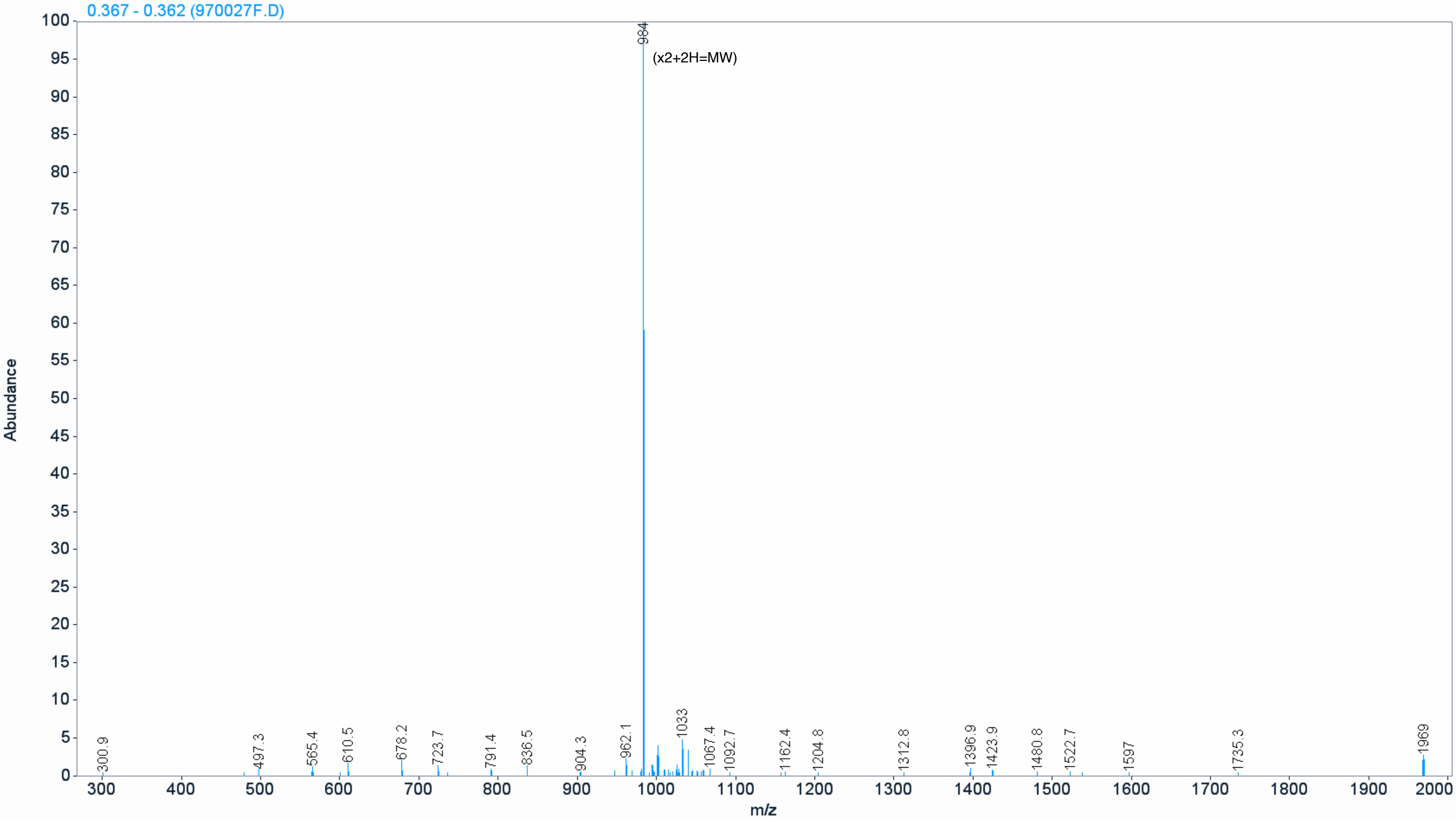

QC files for MP-770

CHINESE PEPTIDE

Certificate of Analysis

**Product Name:**	DMPI-delta (5 to 12 staple)	**Barcode#:**	0952506384<br>0152555212
**Product #:**	973091	**Lot #:**	CQ-03-02804
**Sequence:**	H-DThr-DAla-DTrp(6-F)-DTyr-S8-DAsn-DPhe(4-CF3)-DGlu<br>-DLys-DLeu-DLeu-R5-NH2 (olefin staple from S8 to R5)		
**Molecular Weight:**	1661.9		
**Mass Spectral Analysis:**	Electrospray “Exhibits correct MW”<br>(cis/trans isomers mixture see attached MS spectrogram)		
**HPLC Analysis:**	Peptide purity: 94.3%<br>(see attached RP-HPLC chromatogram)		
**Solubility:**	1 mg/ml in DMSO		
**Appearance:**	White lyophilized powder		
**Counter Ion:**	Trifluoroacetate		
**Cert. of Analysis Remarks:**			

Remarks: **Not for human use, research purpose only.**

Quality Assurance By: Hawk Zhu **Date: 04/26/17**

Quality Control Department

Chinese Peptide Company
No.69,12 Street,Hangzhou Economic & Technological Development Zone,China 310018
Tel: 86-571-86737011 86737118-8686
Fax: 86-571-86737013
Email: sales@chinesepeptide.com
Website: www.chinesepeptide.com

Data file: C:\HPCHEM\1\DATA\042517\973091F.D Sample Name: Final
Sample ID:973091 Lot#:CQ-03-02804
Mobile Phase:A:0.1% TFA in H2O
B:0.09% TFA in (80%ACN+20%H2O)
Flow:1.0 mL/min 46.0%-56.0% B buffer in 20 min
Column:Phenomenex C18(2) 5u 100A 4.6*150mm A832# Lot:D0100046

QC files for MP-770

Injection Date : 4/25/2017 Location : Vial 4
Sample Name : Final Inj. Vol. : 15 µl
Acq Operator : ZWJ
Acq. Method : Final.M
Analysis Method : C:\HPCHEM\1\METHODS\FINAL.M

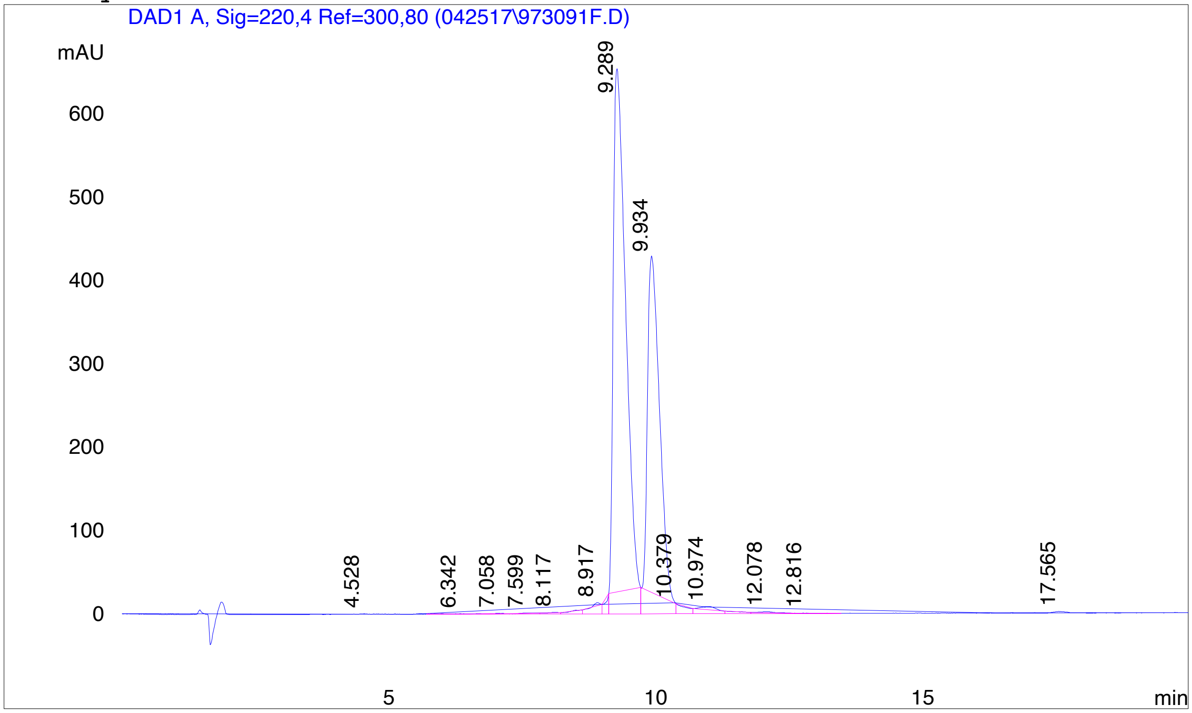

Signal 1:DAD1 A, Sig=220,4 Ref=300,80

Peak #	RT [min]	Type	Height	Width [min]	Area	Area %
1	4.528	BV	0.649	0.160	6.739	0.035
2	6.006	VV	0.518	0.226	7.769	0.041
3	6.342	VV	0.509	0.224	7.253	0.038
4	6.694	VV	0.443	0.174	5.395	0.028
5	7.058	VV	0.874	0.293	17.609	0.092
6	7.599	VV	1.356	0.311	31.691	0.166
7	8.117	VV	1.943	0.266	36.799	0.193
8	8.630	VV	4.812	0.274	79.109	0.414
9	8.917	VV	13.452	0.211	199.111	1.042
10	8.993	VV	11.547	0.160	111.070	0.581
11	9.289	VV	646.180	0.261	10909.525	57.089
12	9.934	VF	427.913	0.264	7117.354	37.244
13	10.379	VV	12.189	0.215	156.887	0.821
14	10.974	VF	8.676	0.404	238.576	1.248
15	11.297	VV	3.246	0.338	65.907	0.345
16	12.078	VV	2.212	0.442	69.253	0.362
17	12.816	VV	0.502	0.226	8.372	0.044
18	13.212	VV	0.412	0.189	5.557	0.029
19	17.565	VV	1.720	0.314	35.847	0.188

*** End of Report ***

QC files for MP-770

973091
Type: Unknown ID: Unknown46 Row: 1
Sample Name:
Sample Name: 973091
Lot #: CQ-03-02804
MW: 1661.9
Sample Type: FINAL
Operator: ZWJ
Instrument Method: D:\data\Method\R&D\MS\MS-290.meth

973091 #9-18 RT: 0.21-0.41 AV: 10 NL: 2.03E8
T: + c ESI Full ms [290.00-2000.00]

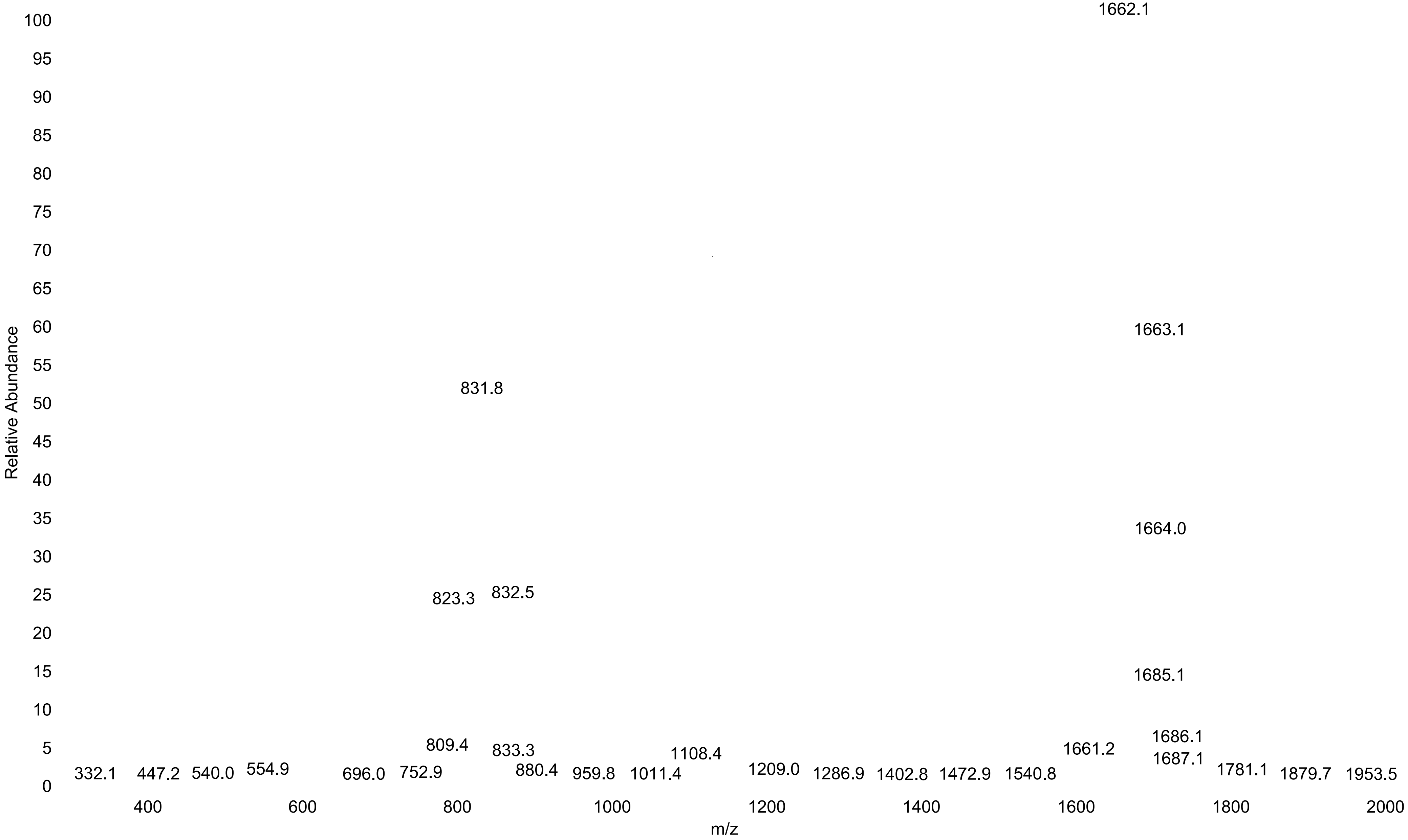

QC files for MP-769

CHINESE PEPTIDE

Certificate of Analysis

**Product Name:**	DMPI-delta (6 to 10 staple)	**Barcode#:**	0952506391<br>0152555311
**Product #:**	973089	**Lot #:**	CQ-03-02802
**Sequence:**	H-DThr-DAla-DTrp(6-F)-DTyr-DAla-R5-DPhe(4-CF3)-DGlu<br>-DLys-R5-DLeu-DArg-NH2 (olefin staple from R5 to R5)		
**Molecular Weight:**	1619.8		
**Mass Spectral Analysis:**	Electrospray "Exhibits correct MW"<br>(see attached MS spectrogram)		
**HPLC Analysis:**	Peptide purity: 94.6%<br>(see attached RP-HPLC chromatogram)		
**Solubility:**	1 mg/ml in DMSO		
**Appearance:**	White lyophilized powder		
**Counter Ion:**	Trifluoroacetate		
**Cert. of Analysis Remarks:**			

Remarks: **Not for human use, research purpose only.**

Quality Assurance By: Hawk Zhu **Date: 04/26/17**

Quality Control Department

Chinese Peptide Company

No.69,12 Street,Hangzhou Economic & Technological Development Zone,China 310018

Tel: 86-571-86737011 86737118-8686

Fax: 86-571-86737013

Email: sales@chinesepeptide.com

Website: www.chinesepeptide.com

Data file: C:\HPCHEM\1\DATA\042517\973089F.D Sample Name: Final
Sample ID:973089 Lot#:CQ-03-02802
Mobile Phase:A:0.1% TFA in H2O
B:0.09% TFA in (80%ACN+20%H2O)
Flow:1.0 mL/min 46.0%-56.0% B buffer in 20 min
Column:Phenomenex C18(2) 5u 100A 4.6*150mm A832# Lot:D0100046

QC files for MP-769

===>

Injection Date : 4/25/2017 Location : Vial 5
Sample Name : Final Inj. Vol. : 10 µl
Acq Operator : ZWJ
Acq. Method : Final.M
Analysis Method : C:\HPCHEM\1\METHODS\FINAL.M

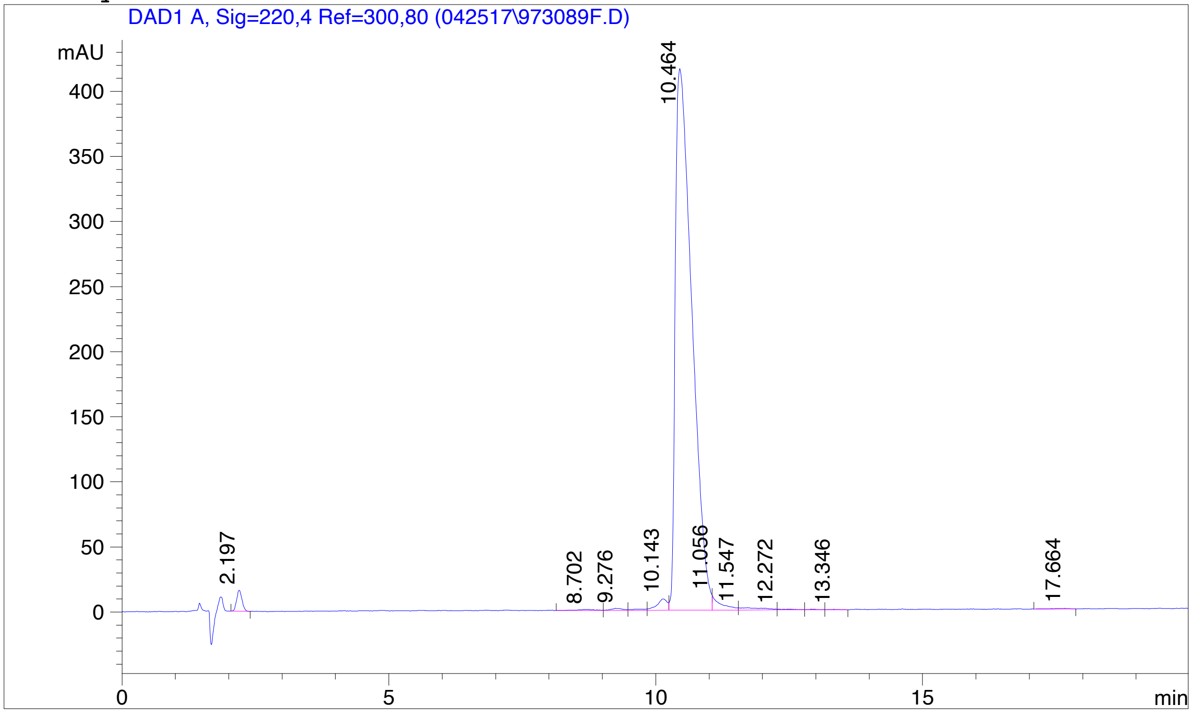

===>

Signal 1:DAD1 A, Sig=220,4 Ref=300,80

Peak #	RT [min]	Type	Height	Width [min]	Area	Area %
1	2.197	MM	16.381	0.115	112.946	1.188
2	8.702	VV	0.846	0.354	22.440	0.236
3	9.276	VV	1.525	0.243	24.168	0.254
4	9.696	VV	0.900	0.274	18.190	0.191
5	10.143	VV	8.686	0.194	115.938	1.219
6	10.464	VF	415.540	0.336	8995.650	94.613
7	11.056	VF	11.398	0.182	124.756	1.312
8	11.547	VF	1.674	0.556	55.888	0.588
9	12.272	VV	0.630	0.352	13.287	0.140
10	12.943	VV	0.374	0.242	6.534	0.069
11	13.346	VV	0.443	0.167	4.896	0.051
12	17.664	MM	0.543	0.403	13.129	0.138

===

*** End of Report ***

\\192.168.15.150\cqcmass\1704\973089F 2017/4/25 12:42:13

QC files for MP-769

973089F
Type: Unknown ID: Unknown46 Row: 1
Sample Name:
Sample Name: 973089
Lot #: CQ-03-02802
MW: 1619.8
Sample Type: FINAL
Operator: ZWJ
Instrument Method: D:\data\Method\R&D\MS\MS-290.meth

973089F #9-20 RT: 0.21-0.45 AV: 12 NL: 2.34E8
T: + c ESI Full ms [290.00-2000.00]

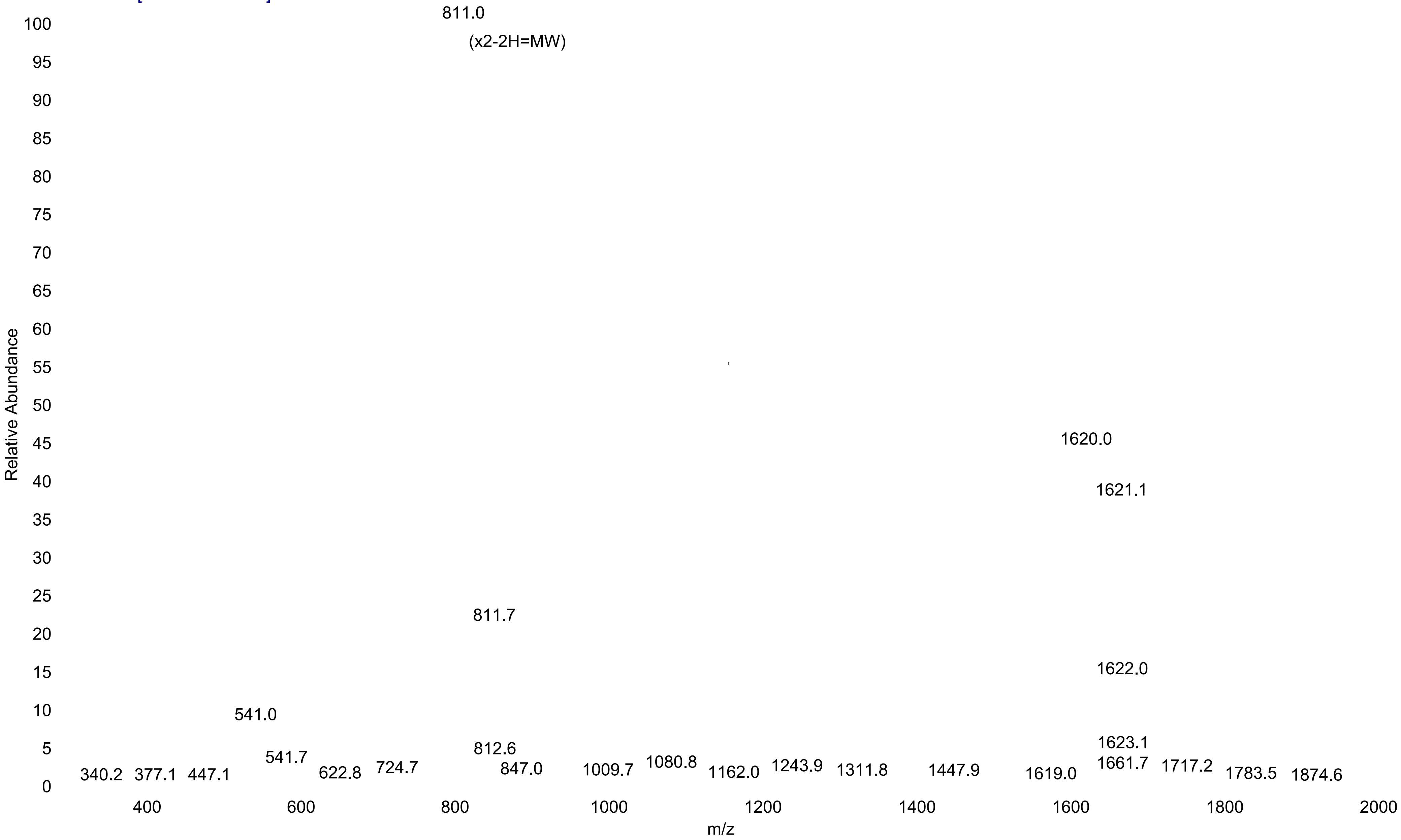

CHINESE PEPTIDE

Certificate of Analysis

**Product Name:**	azide-ATSP-7041 (K1 to E5 lactam)	**Barcode#:**	0952506728 0152555083
**Product #:**	973072	**Lot #:**	CQ-03-02785
**Sequence:**	Ac-Lys(N3)-betaAla-(Lys-Thr-Phe-R8-Glu)-Tyr-Trp-Ala -Gln-Cba-S5-Ser-Ala-Ala-NH2 (olefin staple from R8 to S5, cycle from Glu to Lys)		
**Molecular Weight:**	1967.3		
**Mass Spectral Analysis:**	Electrospray “Exhibits correct MW” (cis/trans isomers mixture see attached MS spectrogram)		
**HPLC Analysis:**	Peptide purity: 94.3% (see attached RP-HPLC chromatogram)		
**Solubility:**	1 mg/ml in DMSO		
**Appearance:**	White lyophilized powder		
**Counter Ion:**	No Salt		
**Cert. of Analysis Remarks:**			

Remarks: **Not for human use, research purpose only.**

Quality Assurance By: Hawk Zhu **Date: 04/20/17**

Quality Control Department

Chinese Peptide Company

No.69,12 Street,Hangzhou Economic & Technological Development Zone,China 310018

Tel: 86-571-86737011 86737118-8686

Fax: 86-571-86737013

Email: sales@chinesepeptide.com

Website: www.chinesepeptide.com

Data file: C:\HPCHEM\1\DATA\041817\973072F.D Sample Name: Final
Sample ID:973072 Lot#:CQ-03-02785
Mobile Phase:A:0.1% TFA in H2O
B:0.09% TFA in (80%ACN+20%H2O)
Flow:1.0 mL/min 65.0%-75.0% B buffer in 20 min
Column:Phenomenex C18(2) 5u 100A 4.6*150mm A832# Lot:D0100046

QC files for MP-002

===->

Injection Date : 4/18/2017 Location : Vial 4
Sample Name : Final Inj. Vol. : 20 µl
Acq Operator : ZWJ
Acq. Method : Final.M
Analysis Method : C:\HPCHEM\1\METHODS\FINAL.M

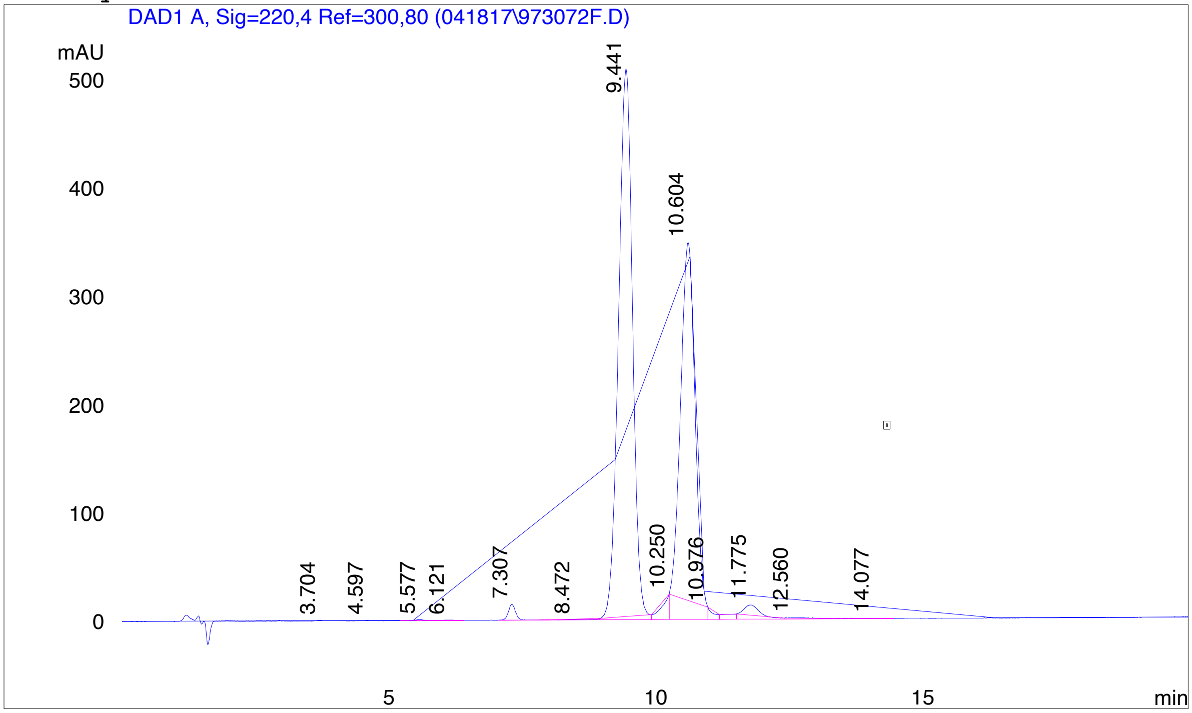

===->

Signal 1:DAD1 A, Sig=220,4 Ref=300,80

Peak #	RT [min]	Type	Height	Width [min]	Area	Area %
1	3.704	VV	0.736	0.239	12.668	0.074
2	4.597	VV	0.425	0.231	6.772	0.040
3	5.577	VV	0.786	0.243	12.956	0.076
4	6.121	VP	0.508	0.281	9.155	0.053
5	7.307	VV	14.752	0.156	140.296	0.820
6	8.472	VV	0.312	0.756	19.530	0.114
7	9.441	VV	508.317	0.284	9313.252	54.422
8	10.250	VV	23.603	0.155	218.902	1.279
9	10.604	VF	348.015	0.306	6838.379	39.960
10	10.976	VV	11.205	0.117	78.457	0.458
11	11.363	VV	4.667	0.259	85.679	0.501
12	11.775	VV	13.046	0.359	315.516	1.844
13	12.560	VV	1.049	0.547	46.845	0.274
14	14.077	VV	0.373	0.482	14.595	0.085

===

*** End of Report ***

\\192.168.15.150\cqcmass\1704\973072F 2017/4/14 15:10:58

QC files for MP-002

973072F
Type: Unknown ID: Unknown Row: 1
Sample Name:
Sample Name: 973072
Lot #: CQ-03-02785
MW: 1967.3
Sample Type: Final
Operator: JJK
Instrument Method: D:\data\Method\R&D\MS\MS-290.meth

973072F #4-10 RT: 0.08-0.22 AV: 7 NL: 5.20E7
T: + c ESI Full ms [290.00-2000.00]

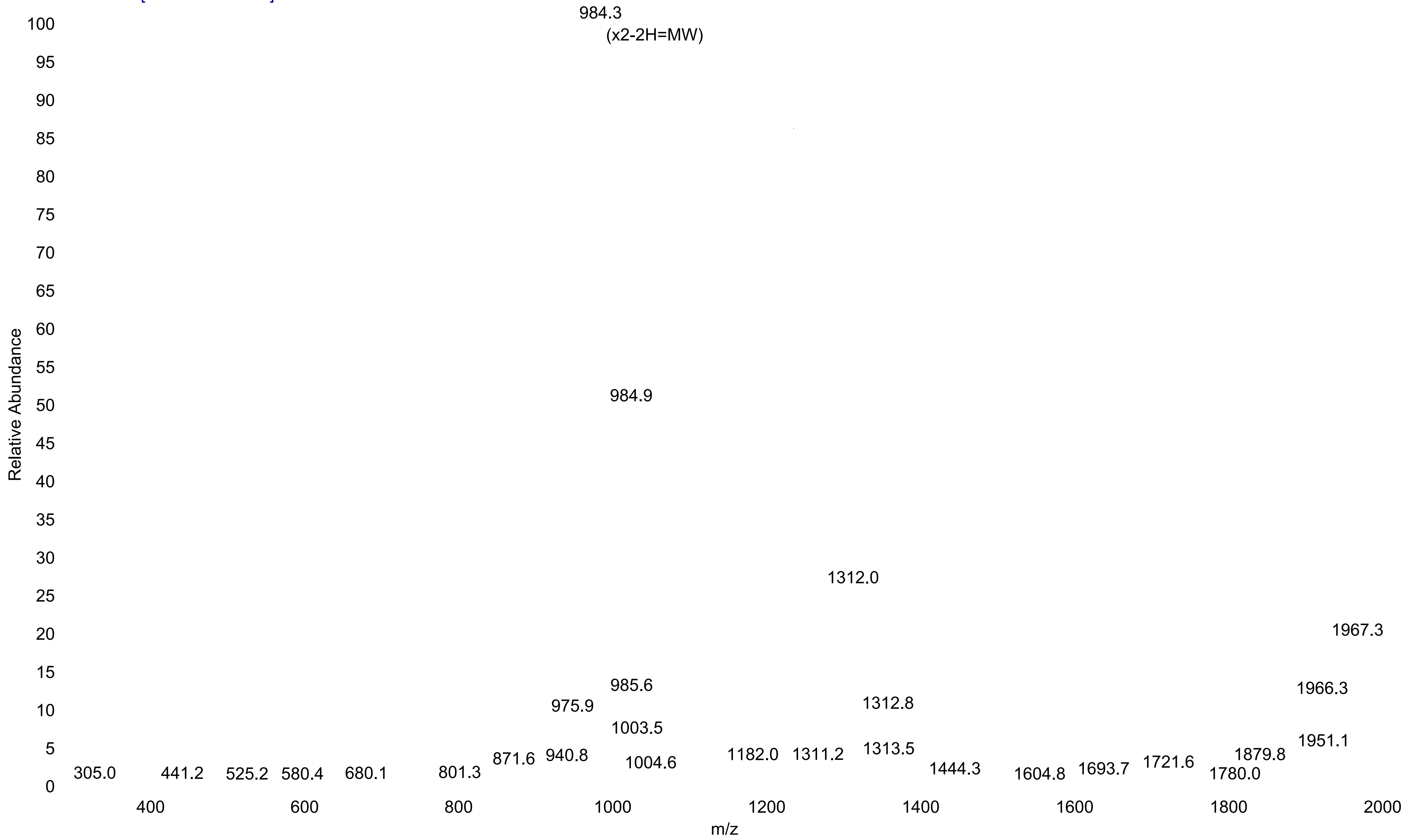

QC files for MP-950

CHINESE PEPTIDE

Certificate of Analysis

**Product Name:**	EEE-azide-SGS-PM2-EEE	**Barcode#:**	0955005020<br>0154300353
**Product #:**	975714	**Lot #:**	CT-04-00695

Sequence: Glu-Glu-Glu-Lys(N3)-Ser-Gly-Ser-Thr-Ser-Phe-R8-Glu-Tyr-Trp -Ala-Leu-Leu-S5-Glu-Glu-Glu-NH2 (olefin staple from R8 to S5)

Molecular Weight: 2580.8

Mass Spectral Analysis: Electrospray “Exhibits correct MW” (see attached MS spectrogram)

HPLC Analysis: Peptide purity: 93.4% (cis/trans isomers mixture see attached RP-HPLC chromatogram)

Solubility: 1 mg/ml in DMSO

Appearance: Off-white lyophilized powder

Counter Ion: Trifluoroacetate

Cert. of Analysis Remarks:

Remarks: **Not for human use, research purpose only.**

Quality Assurance By: Hawk Zhu **Date: 05/15/20**
Quality Control Department

Chinese Peptide Company
No.69,12 Street,Hangzhou Economic & Technological Development Zone,China 310018
Tel: 86-571-86737011 86737118-8686
Fax: 86-571-86737013
Email: sales@chinesepeptide.com
Website: www.chinesepeptide.com

Data file:C:\HPCHEM\1\DATA\051420\975714F.D Sample Name:FINAL
Sample ID:975714 Lot:CT-04-00695
Mobile phase:A:0.1% TFA in H2O
B:0.09% TFA in (80%ACN+20%H2O)
Flow:1.0 ml/min 50.0%-60.0% B buffer in 20min
Column:Phenomenex C18(2) 5u 100A 4.6*150mm A1112#

QC files for MP-950

===>

Injection Date : 5/14/2020 Location : Vial 92
Sample Name : Final Inj. Vol. : 5 µl
Acq Operator : ZWJ
Acq. Method : Final.M
Analysis Method : C:\HPCHEM\1\METHODS\FINAL.M

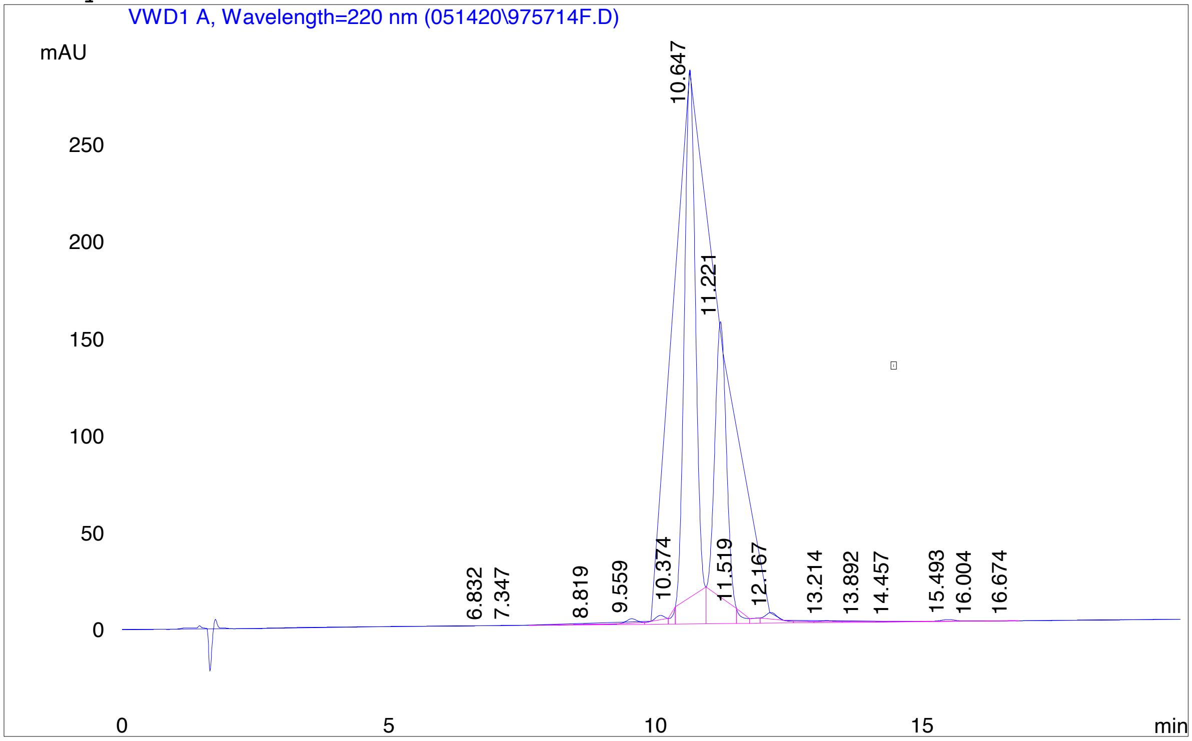

===>

Signal 1:VWD1 A, Wavelength=220 nm

Peak #	RT [min]	Type	Width [min]	Height	Area	Area %
1	6.832	VV	0.237	0.107	1.643	0.022
2	7.347	VV	0.362	0.097	2.283	0.031
3	8.819	VV	0.596	0.388	17.303	0.236
4	9.559	VV	0.267	2.923	52.371	0.716
5	10.099	VV	0.247	4.510	75.215	1.028
6	10.374	VV	0.065	8.905	34.606	0.473
7	10.647	VV	0.240	285.072	4300.078	58.767
8	11.221	VF	0.255	155.240	2539.314	34.704
9	11.519	VV	0.114	7.673	52.365	0.716
10	11.966	VV	0.192	2.728	31.407	0.429
11	12.167	VV	0.289	5.493	108.672	1.485
12	12.753	VV	0.304	0.836	17.616	0.241
13	13.214	VV	0.333	1.002	23.078	0.315
14	13.892	VV	0.510	0.583	22.607	0.309
15	14.457	VV	0.342	0.337	7.710	0.105
16	15.493	VV	0.375	1.007	26.300	0.359
17	16.004	VV	0.203	0.114	1.593	0.022
18	16.674	VV	0.444	0.088	2.948	0.040

===

*** End of Report ***

QC files for MP-950

Data file: D:\DATE\200514\20200514-1 2020-05-14 11-30-26\975714.D 5/14/2020 12:44:17 PM

Sample name: 975714

Description: Lot#:CT-04-00695
MW:2580.8
Sample Type:Final

Acq. method: 290-2000-Negative-
Frag 130v.M

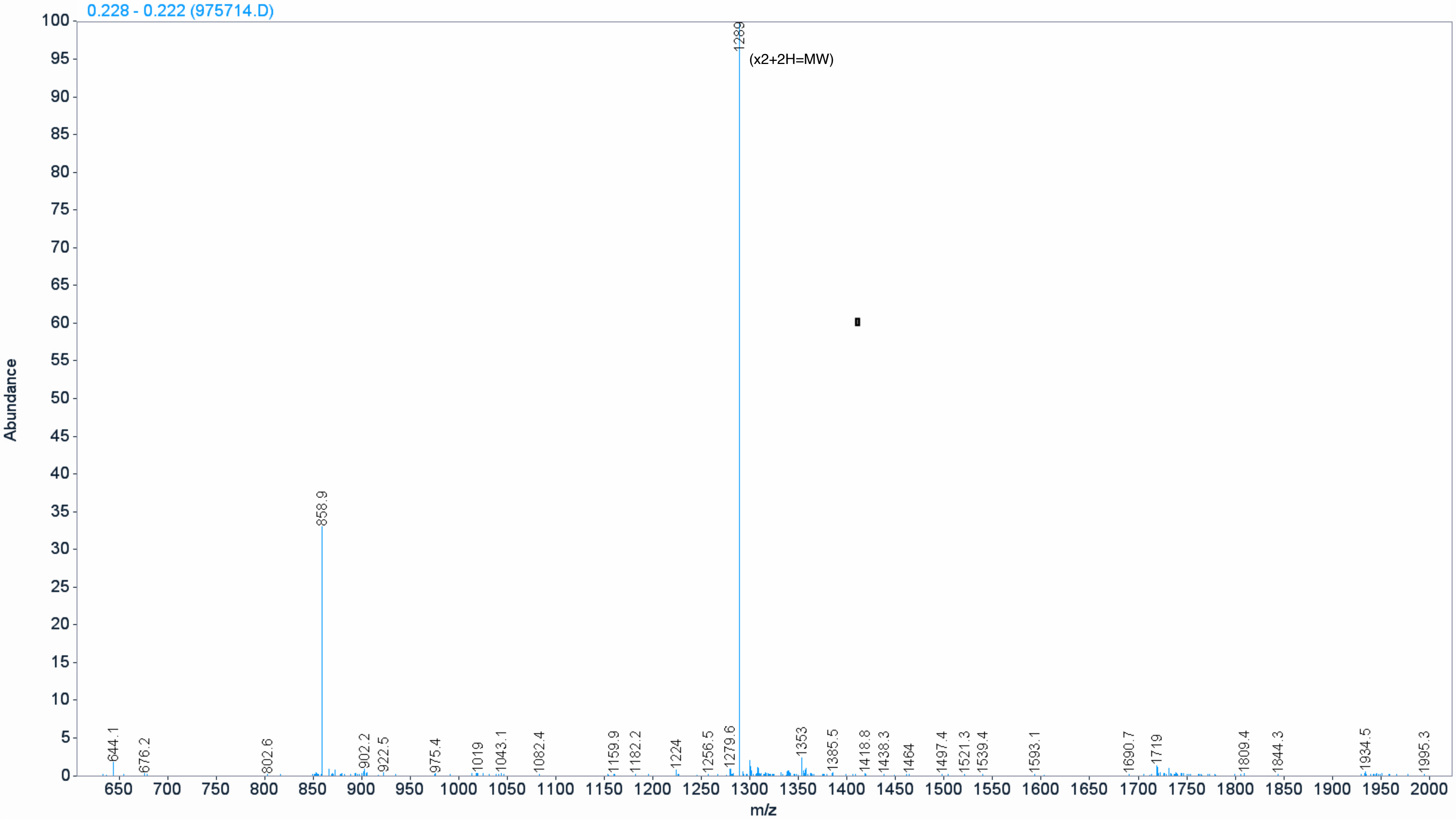

```
Data file:C:\HPCHEM\1\DATA\062921\994408F.D      Sample Name:FINAL
Sample ID:994408     Lot:CU-06-00367
Mobile phase:A:0.1% TFA in H2O
             B:0.09% TFA in (80%ACN+20%H2O)
Flow:1.0 ml/min        58.0%-68.0% B buffer in 20 min
Column:Phenomenex C18(2) 5u 100A 4.6*150mm A1184#
Equipment ID:2014070201  Computer ID:C6307082701                    ->
=====================================================================->
Injection Date  : 6/29/2021                       Location   :     Vial 22
Sample Name     : Final                           Inj. Vol.  :        5 µl
Acq Operator    : ZWJ
Acq. Method     : Final.M
Analysis Method : C:\HPCHEM\1\METHODS\FINAL.M
```

QC files for MP-950

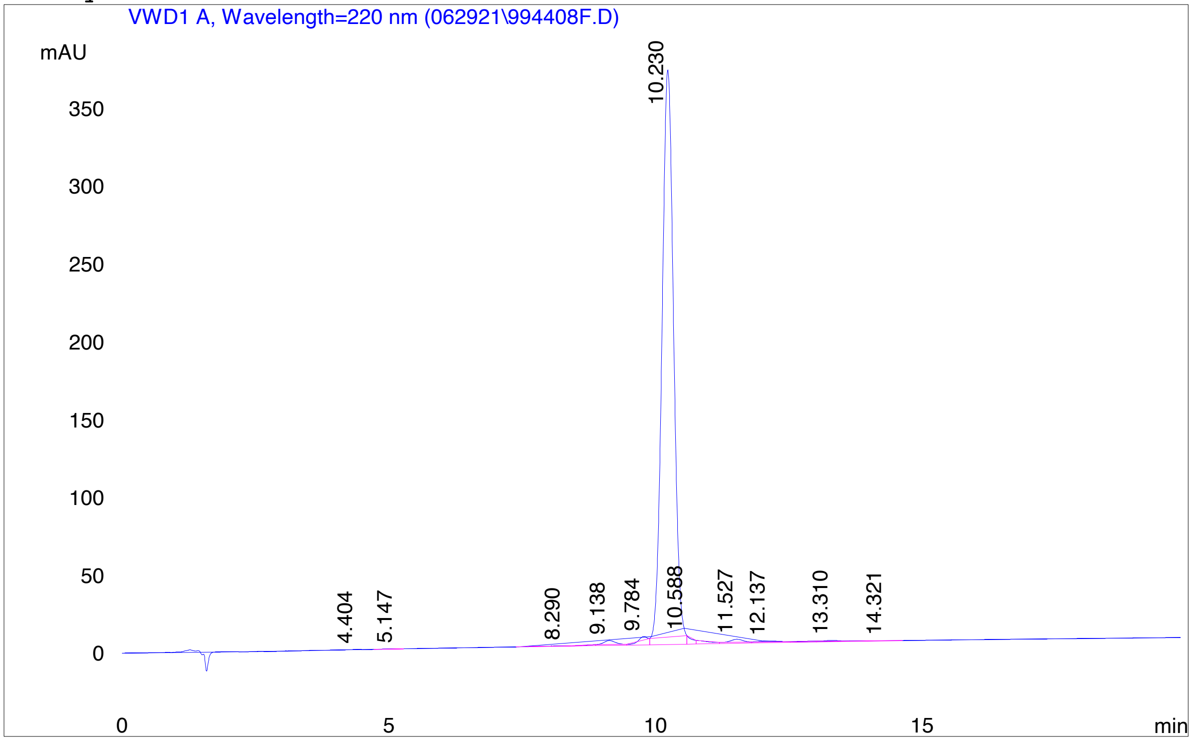

===->

Signal 1:VWD1 A, Wavelength=220 nm

Peak #	RT [min]	Type	Width [min]	Height	Area	Area %
1	4.404	BV	0.344	0.315	8.190	0.136
2	5.147	VV	0.282	0.220	4.217	0.070
3	8.290	VV	0.479	0.275	10.214	0.170
4	8.879	VV	0.265	0.701	11.143	0.186
5	9.138	VV	0.272	3.140	57.575	0.960
6	9.784	VV	0.197	5.325	71.245	1.187
7	10.230	VF	0.236	368.406	5622.391	93.705
8	10.588	VF	0.105	5.563	35.171	0.586
9	10.762	VV	0.262	2.356	37.096	0.618
10	11.527	VV	0.306	2.610	54.162	0.903
11	12.137	VV	0.490	1.166	39.122	0.652
12	13.014	VV	0.502	0.558	16.793	0.280
13	13.310	VV	0.361	0.916	22.837	0.381
14	14.321	VV	0.812	0.159	9.931	0.166

===

*** End of Report ***
