# Supplementary material for: Design-rules for stapled peptides with in vivo activity and their application to Mdm2/X antagonists
Source: Nat Commun. 2024 Jan 12;15:489. doi: 10.1038/s41467-023-43346-4 (PMC10786919; doi:10.1038/s41467-023-43346-4)
Supplement: Supplementary file 3 — Description of Additional Supplementary files [file 41467_2023_43346_MOESM3_ESM.pdf]

Description of Additional Supplementary Items

File name: Supplementary Data 1
Description: Supplementary Table 1: Peptide unique identifiers (MP-IDs), sequences (chemical name), peptide names (trivial name), and processed data for all peptides synthesized in the current study.
